# Application of AI and digital health tools in public health management of T2DM: from mechanism prediction to personalized treatment

**DOI:** 10.3389/fpubh.2026.1756755

**Published:** 2026-02-17

**Authors:** Chonger Yu

**Affiliations:** Beijing University of Chinese Medicine, Beijing, China

**Keywords:** artificial intelligence, data privacy, digital health, personalized medicine, public health management, type 2 diabetes mellitus

## Abstract

Type 2 diabetes mellitus (T2DM) poses a significant global public health challenge, with its prevalence escalating continuously and disproportionately affecting low- and middle-income countries (LMICs), imposing a substantial burden on healthcare systems. Traditional management models have limitations in disease prediction, personalized treatment, and public health intervention. Artificial intelligence (AI) and digital health technologies provide novel insights for precise prediction and intelligent management of T2DM. This review systematically summarizes research progress in AI’s role in deciphering T2DM pathogenesis, personalized treatment, and public health management. By integrating multi-omics and environmental data, AI reveals key mechanisms including gene–environment (G × E) interactions, *β*-cell dysfunction, and inflammatory pathways, significantly enhancing early screening and risk prediction. In clinical management, AI combined with digital health tools [e.g., continuous glucose monitoring (CGM), wearable devices, and mobile health (mHealth) apps] facilitates remote monitoring, medication optimization, and personalized interventions, improving treatment adherence and health management efficiency. At the public health level, AI optimizes resource allocation and disease burden assessment, promoting chronic disease prevention and control model transformation. Future efforts should prioritize developing low-resource-adapted tools, strengthening data privacy protection tailored to LMICs, and addressing algorithmic fairness and the digital divide to ensure safe, equitable, and sustainable AI application in global T2DM management. Overall, AI and digital health integration is driving T2DM management towards an intelligent and precision-based era, with the potential to reduce disparities in LMICs.

## 1 Introduction

The global incidence and prevalence of T2DM have experienced explosive growth over the past few decades. Approximately 537 million people worldwide had diabetes in 2021, and this number is projected to rise to 783 million by 2045 (1). T2DM accounts for 90% of all cases (2), and nearly 80% occur in LMICs, where limited healthcare resources, weak digital infrastructure, and socioeconomic constraints exacerbate the disease burden (3). For instance, China, the world’s largest diabetic population, has around 140 million individuals with diabetes (mostly T2DM) (4). Data from the Global Burden of Disease (GBD) study further indicate that the burden of T2DM has continued to increase from 1990 to 2021, particularly among low-income rural populations and women of childbearing age (5). Certain regions, such as South Asia and sub-Saharan Africa, bear a particularly heavy burden of T2DM, with incidence expected to continue rising by 2031 (6). Meanwhile, T2DM and its complications impose a severe burden on LMIC healthcare systems, which are already strained by resource shortages. In Indonesia, in-hospital mortality among T2DM patients is 2–3 times higher than in high-income countries (HICs) due to delayed diagnosis and limited access to glucose monitoring tools (7). In sub-Saharan Africa, the economic burden of T2DM consumes 15–20% of national health budgets, diverting funds from infectious disease control (8). To address this global public health challenge, various context-adapted interventions are being explored, including evaluating the effectiveness of the Patient-Centered Medical Home (PCMH) model (9), applying mHealth interventions for T2DM prevention and control in low-income countries like Bangladesh (3), and enhancing patients’ self-management capabilities within the framework of community public health nursing in Kenya (4).

The COVID-19 pandemic has strained global healthcare systems, with substantial resources diverted to pandemic response, severely disrupting routine T2DM care. Firstly, non-urgent outpatient services were suspended/reduced, hindering regular follow-ups, blood glucose monitoring, medication adjustments, and complication screening required by T2DM patients. This easily leads to poor blood glucose control and increased risks of acute and chronic complications. Moreover, admission hyperglycemia is the best predictor of SARS-CoV-2 imaging findings (10), highlighting the importance of standardized T2DM management during the pandemic. Secondly, shortages of hospital beds and healthcare personnel were exacerbated by T2DM patients with comorbid COVID-19, who tend to have more severe conditions, longer hospital stays, higher Intensive Care Unit (ICU) requirements, and significantly increased risks of in-hospital hyperglycemia and associated mortality. Thirdly, healthcare workers suffered from physical and mental exhaustion due to long-term high-intensity work, infection risks, and ethical dilemmas, affecting both pandemic treatment and the management of chronic diseases like T2DM.

Faced with shortages of medical resources and risks associated with in-person consultations, telemedicine has become an important alternative for T2DM management, with a significant surge in demand. Leveraging telephone, video, mobile applications, and wearable devices, it enables remote medical consultation and guidance for patients. On one hand, the pandemic accelerated the popularization of digital health tools, such as remote blood glucose monitoring systems and mHealth applications. Combined with the PCMH model, which had demonstrated potential before the pandemic (9), it provides T2DM patients with real-time diagnosis and treatment adjustments, self-management guidance, and coordinated care. On the other hand, telemedicine breaks geographical barriers, improving healthcare accessibility for patients in remote areas and those with mobility impairments (9), particularly benefiting individuals facing difficulties in seeking medical care during pandemic lockdowns. Despite its crucial role, telemedicine still faces challenges such as the digital divide, data privacy and security concerns, and inadequate reimbursement policies. Meanwhile, the pandemic has brought opportunities for technological innovation, policy optimization, and infrastructure improvement, positioning telemedicine as a core component of T2DM management in the post-pandemic era.

T2DM has become a major public health issue requiring urgent global attention, with its rising prevalence exerting profound impacts on healthcare systems and socioeconomic development. Although traditional management models have played a certain role in disease prediction, personalized treatment, and public health intervention, their limitations have become increasingly apparent. In recent years, the rapid development of AI and digital health tools has provided new opportunities for the public health management of T2DM, promising to drive a paradigm shift in disease prediction, prevention, and treatment, and demonstrating great potential in improving overall health management (11).

This review aims to explore the application of AI and digital health tools in the public health management of T2DM, focusing on their roles in disease mechanism prediction and personalized treatment, and further analyzing their integration pathways with public health policies. First, it reviews the global epidemiological characteristics of T2DM, highlighting the challenges posed to public health systems and the shortcomings of existing management strategies. Second, it elaborates on the application prospects of AI in predicting pathogenesis, including identifying biomarkers and key pathogenic pathways based on multi-omics data, and constructing disease progression models using electronic health records (EHRs) and wearable device data, thereby providing a theoretical and practical basis for early warning and intervention. Subsequently, it systematically analyzes the potential of AI and digital health tools in personalized treatment, covering AI-based drug selection and dosage optimization, as well as the role of intelligent blood glucose management systems and mHealth applications in precise self-management. Finally, from an interdisciplinary perspective, it discusses the connection and interaction between technological innovation and public health policies, emphasizing the importance of establishing a regulatory framework centered on data privacy protection and algorithmic fairness, and proposing the need to promote equitable and accessible digital health policies to narrow the digital divide. In summary, this review intends to provide a systematic reference for policymakers, researchers, and clinicians, facilitating the full utilization of AI and digital health tools in the public health management of T2DM to address the growing disease burden.

## 2 Application of AI in predicting the pathogenesis of T2DM

### 2.1 G × E interaction models

Combined with big data analytics, AI has demonstrated significant advantages in constructing T2DM prediction models, aiming to achieve early screening, health management, and personalized prevention through accurate identification of risk factors.

#### 2.1.1 Core role of AI and big data in T2DM prediction

T2DM is a complex metabolic disease influenced by both genetic and environmental factors. Traditional statistical methods struggle to handle high-dimensional multimodal data, while AI combined with big data analytics can overcome this limitation, providing new approaches for risk prediction. AI technologies are not merely abstract tools but are tailored to T2DM’s multi-factor pathogenesis—machine learning (ML) and deep learning (DL) algorithms extract disease-specific patterns from massive datasets, directly improving the accuracy of T2DM risk stratification and early screening (12). T2DM prediction involves genomic data (e.g., single-nucleotide polymorphisms, SNPs), medical imaging, clinical indicators, lifestyle, and environmental exposure information (13–16). Big data analytics excels at processing these heterogeneous high-dimensional datasets. It provides abundant high-quality inputs for AI model training, laying a solid foundation for accurate risk stratification. Meanwhile, AI-based diabetes prediction models can efficiently handle large volumes of disease data from detailed patient information and diagnostic reports (17).

Common ML algorithms include decision tree (DT, a tree-like classification model that recursively splits data based on clear decision rules, e.g., “if fasting blood glucose >7mmol/L and BMI >28 kg/m^2^, classify as high-risk”—easy to interpret for non-technical readers), Random Forests (RF, an ensemble algorithm integrating multiple independent DTs, reducing overfitting by “majority voting” of individual tree predictions, suitable for noisy clinical data), logistic regression (LR, a statistical model that calculates T2DM onset probability via a linear combination of features like age, blood pressure, and lifestyle), support vector machines (SVM, a model that finds the optimal “separating line” in high-dimensional space to distinguish high/low-risk groups, effective for small-scale but high-quality datasets), and eXtreme Gradient Boosting (XGBoost, an optimized gradient boosting algorithm that iteratively trains weak models to correct prediction errors, emphasizing key features like genetic variants and dietary patterns, and handling missing data robustly—ideal for T2DM’s multi-factor pathogenesis). These have been used to construct T2DM risk assessment models capable of effectively distinguishing between patients and healthy controls (18). XGBoost, optimized for T2DM’s G × E interactions, has been prospectively validated in a Han Chinese cohort (*n* = 10,232) over 5 years, achieving an Area Under the ROC Curve (AUC) of 0.78 for 5-year T2DM incidence prediction—30% lower false negatives than traditional methods, directly addressing T2DM’s missed diagnosis problem in early screening. Models integrating XGBoost and Genetic Risk Scores (GRS) reduced incident T2DM by 18% in a randomized controlled trial (RCT) via targeted lifestyle interventions, with no increase in hypoglycemia risk (19). As shown in Table 1, XGBoost has been scaled in primary care settings in China (covering over 500,000 individuals) due to low computational requirements, though its generalizability across populations remains limited—failing cross-ethnic validation in rural India with an AUC dropping to 0.59 (13). Additionally, studies have proposed novel models based on data mining techniques to improve prediction accuracy and adapt to multiple datasets (11).

**Table 1:** Summary table of current status of AI technology clinical implementation.

AI technology	Prospective validation evidence	Clinical benefit	Scalability status	Failures/limitations
XGBoost	Han Chinese cohort (*n* = 10,232), 5-year AUC = 0.78(13)	18% reduction in incident T2DM; 0.5% HbA1c reduction (19)	Scaled in China (500 k + individuals); limited in LMICs	Cross-ethnic validation failure (AUC = 0.59 in India) (24)
DL models	European cohort (*n* = 5,000), AUC = 0.82 for incidence prediction (21)	15% TIR increase; no hypoglycemia increase (23)	Not scalable in LMICs; academic use only	Failed in rural India due to environmental data gaps (24)
FL	Multi-center EU study (5 hospitals), AUC = 0.76(105)	0.7% HbA1c reduction; zero data breaches (105)	Scaled in EU; failed in LMICs (bandwidth gaps)	15–20% accuracy loss vs. centralized training (137)
Digital twins	RCT (*n* = 150), 85% accuracy in hypoglycemia prediction (75)	35% reduction in severe hypoglycemia (75)	Limited to academic centers	Failed in older adult comorbid patients (accuracy <60%) (75)
Edge AI	Rural China (*n* = 200 k), AUC = 0.71 for screening (106)	40% reduction in diagnostic delays (106)	Scaled in rural China; scalable in LMICs	No major failures; limited to screening (no treatment optimization)

DL algorithms include two key subtypes with distinct clinical applications. One is Convolutional Neural Networks (CNNs), which specialize in extracting spatial features from medical images (e.g., retinal fundus photos) to predict complications like diabetic retinopathy. The other is Recurrent Neural Networks (RNNs), designed for time-series data such as 72-h CGM glucose fluctuations. These DL algorithms excel at capturing complex biological patterns in T2DM-related data. Unlike generic pattern recognition, they analyze genomic variations and clinical phenotypes specific to insulin resistance and *β*-cell dysfunction (20, 21). For example, a CNN-based model can automatically identify microaneurysms in retinal images (early diabetic retinopathy) by learning hierarchical visual features, while an RNN can predict postprandial glucose spikes by recognizing temporal correlations between meal intake, physical activity, and glucose trends (22). As summarized in Table 1, DL models have demonstrated promising performance in European cohorts (AUC = 0.82 for incidence prediction) (21), achieving a 15% increase in TIR without increasing hypoglycemia risk (23). However, they failed prospective validation in rural India (AUC dropped from 0.82 to 0.59) due to unaccounted environmental factors (e.g., malnutrition) (24), and their scalability in LMICs remains restricted to academic use only.

#### 2.1.2 Integrated analysis of genetic and environmental factors

T2DM exhibits significant genetic susceptibility. Studies have analyzed SNPs loci through genotyping (such as polymerase chain reaction–ligase detection reaction) and quantified individual genetic susceptibility using GRS, constructing early prediction models integrating GRS and non-GRS for the Han Chinese population (25). Polymorphisms in the ATP-binding cassette transporter subfamily a member 1 (ABCA1) gene may also be associated with T2DM risk (26), and AI can mine potential correlations from large-scale genetic data to assist in proposing and verifying genetic hypotheses (27).

Lifestyle factors (dietary habits, physical activity), environmental exposures (e.g., metal exposure), and socioeconomic factors all play important roles in the occurrence and development of T2DM (14–16). Research has found that multiple environmental factors may interact with individual genetic factors to influence disease phenotypes such as Body Mass Index (BMI). By genotyping and performing quality control of samples from the Korean Association Resource (KARE) dataset, excluding samples with missing data, this study ultimately tested the interaction between environmental factors and SNPs in 8155 samples to identify potential SNPs associated with BMI (15).

However, ethical dilemmas in G × E interaction modeling are increasingly prominent—with unique complexities in LMICs. First, genetic data used in G × E models is inherently sensitive, but LMICs often lack dedicated regulations to protect it: Only 20% of LMICs have laws governing genetic privacy (24), leaving patients vulnerable to discrimination (e.g., employers denying jobs based on predicted T2DM risk) (28).

Second, informed consent for genetic data sharing is often inadequate in LMICs due to low health literacy: 60–70% of participants in rural LMIC cohorts do not fully understand the long-term implications of disclosing genetic information, leading to “token consent” without true autonomy (29).

Third, G × E models trained on HIC data risk overemphasizing genetic determinism in LMICs, downplaying structural barriers (e.g., food insecurity and lack of clean water) and shifting blame to individuals for their disease risk (30). For example, a G × E model developed in Europe underestimated T2DM risk in rural Ghana by 35% because it failed to account for environmental stressors like malnutrition and limited physical activity opportunities (31). This ethical bias is exacerbated by the lack of LMICs representation in global genetic databases—over 90% of T2DM genetic research data comes from HICs, despite LMICs accounting for most cases (24).

While AI can simultaneously analyze the complex interactions between genetic and environmental factors [e.g., the XGBoost model developed T2DM prediction models based on genetic and medical imaging data (13); transfer learning frameworks can enhance the modeling capability of small-scale paired data using large-scale unpaired clinical data to improve risk prediction when multimodal data is scarce (22)], current cross-population G × E interaction studies face methodological and application limitations: Most rely on single-population cohorts (predominantly of European ancestry), lacking genetic background diversity and failing to capture heterogeneity in allele frequencies, linkage disequilibrium structures, and environmental exposures among different ethnic groups. For example, polymorphisms in the Transcription Factor 7 Like 2 (TCF7L2) gene are associated with T2DM risk in rural Chinese cohorts and regulated by folic acid intake (19), but their effect size may differ in Indian and African populations, and existing models rarely undergo cross-population validation—restricting model generalizability and clinical translation.

Future efforts should establish large-scale, multi-center, longitudinally followed diverse-population cohorts and develop more interpretable and robust AI analysis paradigms to address these gaps.

#### 2.1.3 Construction and optimization of T2DM prediction models

Screening features with high predictive value from clinical and genetic data is crucial for constructing efficient models. Key genetic variants can be screened using methods such as Least Absolute Shrinkage and Selection Operator (LASSO) and Recursive Feature Elimination (RFE), and ML methods can also be applied for this purpose to improve prediction accuracy (32). For example, LASSO feature selection combined with RF was prospectively validated in the KARE dataset (*n* = 8,155), predicting BMI-related T2DM risk with consistent performance over 3 years (AUC = 0.72). This model identified high-risk individuals who, when enrolled in personalized diet interventions, achieved a 0.5% reduction in glycated hemoglobin (HbA1c, a key indicator of long-term blood glucose control) and a 12% increase in time-in-range (TIR, 70–180 mg/dL) vs. usual care (14)—key performance metrics that align with the core applications of AI in T2DM pathogenesis prediction as outlined in Table 2. Additionally, model training often adopts cross-validation to ensure generalizability and robustness.

**Table 2:** Summary of AI applications in predicting T2DM pathogenesis.

Core module	Specific direction	Key content points
G × E interaction models	Core role of AI and big data	Break through the limitations of traditional statistics to handle high-dimensional data. ML and DL improve prediction accuracy. Cover multiple types of data, with big data technology supporting heterogeneous data processing.
	Integrated analysis of genetic and environmental factors	Genetics: Genotyping analyzes SNPs, GRS quantifies susceptibility, AI mines gene associations. Environment: Lifestyle and other factors interact with genetics to affect phenotypes such as BMI. Limitation: Few cross-population G × E studies, models mostly rely on European populations.
	Model construction and optimization	Key steps: LASSO for feature selection, cross-validation to ensure generalizability. Applications: Personalized risk assessment, complication prediction. Challenges: “Black box” nature of models, data privacy limits clinical adoption, need to strengthen XAI application.
Simulating *β*-cell dysfunction and inflammatory pathways	Core pathological mechanisms	β-cell dysfunction: Oxidative/endoplasmic reticulum stress and chronic inflammation lead to insufficient insulin secretion after compensating for insulin resistance. Systemic inflammation: Elevated levels of markers such as IL-6 and IL-1β, TLR4/NFκB pathway exacerbate β-cell damage. The two form a vicious cycle, with PAK family as key targets.
	Core applications of AI	Prediction and diagnosis: Use EHRs and inflammatory indicators for risk stratification. Mechanism simulation: Process multi-omics data, Boolean networks simulate β-cell apoptosis, fMRI identifies cognitive impairment associations. Drug discovery: Identify targets, optimize insulin therapy, analyze traditional Chinese medicine mechanisms.
	Existing challenges	Data: Need higher-quality multi-omics/longitudinal datasets. Models and applications: Insufficient interpretability, need to develop multi-level integration models and promote personalized plans. Emerging directions: Combine wearable devices for real-time monitoring, explore associations with neurodegenerative diseases.
AI-based integration of multi-omics data	Application value of multi-omics data	Genomics: Identify T2DM-susceptible SNPs through GWAS, need to combine other omics to explain complexity. Metabolomics: Analyze changes in metabolites such as blood glucose/lipids, which can serve as early diagnostic biomarkers. Microbiomics: Gut microbiota dysbiosis is associated with insulin resistance, oral microbiota (such as in periodontitis patients) may be involved in pathological processes.
	AI integration methodologies	Data processing: Use PCA/tSNE for dimensionality reduction and Lasso for feature selection to address high-dimensional/heterogeneous data challenges. Algorithm application: Tree-based boosting methods improve prediction accuracy, DL mines complex patterns, semi-supervised/unsupervised learning for patient classification.
	Application value and challenges	Value: Construct mechanism maps for early prediction, assist personalized treatment, combine CGM to optimize blood glucose management. Challenges: Difficult data standardization, unclear model causal relationships, need for large-scale cohorts, address clinical translation and ethical issues.

However, overfitting remains a pervasive challenge in AI-driven T2DM prediction models, particularly in G × E and multi-omics integration. Overfitting occurs when models memorize noise in training data (e.g., small sample sizes and redundant features) rather than learning generalizable patterns—for instance, a DL model trained on 500 patients may achieve 95% accuracy in the training set but drop to 65% in external validation (22). This not only compromises clinical utility but also exacerbates health disparities: Models overfitted to homogeneous cohorts (e.g., young urban adults) perform poorly in underrepresented groups (e.g., older adult rural populations) (33). Although cross-validation and LASSO feature selection can mitigate this risk, they cannot fully eliminate it—especially when data is scarce (a common issue in LMICs), leading to overreliance on noisy or biased features (34).

Meanwhile, differences in population genetic backgrounds and environments lead to poor cross-population applicability of models. Future research should develop models with high generalizability or explore population-specific strategies. Moreover, multi-source data (genes, multi-omics, gut microbiota, wearable devices, imaging, environment, etc.) can be integrated into EHRs as multimodal big data for utilization by AI and its subfields (ML, DL). Different ML types, such as supervised, semi-supervised, unsupervised, and reinforcement learning (RL), can all assist in medical big data analysis and clinical decision-making (35). Currently, such integration is ready for population-scale deployment in countries with centralized EHRs (e.g., South Korea and Singapore), but only 15% of LMICs have the infrastructure to support it (34).

In terms of personalized prediction, AI models can achieve personalized T2DM risk assessment by incorporating individual differences, such as personalized T2DM prediction models that integrate clinical, behavioral, and dietary data (including glycemic index and glycemic load) (36), as well as ML-based personalized prediction models for urinary tract infections in T2DM patients (37). In terms of model interpretability and clinical application, as the complexity of AI models increases, the “black box” nature of AI decision-making processes has become a challenge. Explainable artificial intelligence (XAI) can reveal the genetic insights behind predictions, enhance clinical trust, and provide clues for disease mechanism research. However, the opaque internal working mechanisms of models, combined with interpretability and data privacy issues, limit clinicians’ trust and adoption of their prediction results (17, 21). Future efforts should strengthen the application of XAI to reveal the biological mechanisms behind model decisions.

In summary, AI combined with big data analytics of genetic and environmental factors provides a new paradigm for T2DM prediction and management. Nevertheless, it still faces data challenges and model interpretability issues. Future research should develop emerging AI technologies, accumulate clinical data, improve model accuracy and generalizability, and contribute to disease prevention and personalized medicine.

### 2.2 Simulating *β*-cell dysfunction and inflammatory pathways

T2DM is a complex metabolic disorder characterized by insulin resistance and pancreatic *β*-cell dysfunction, with chronic inflammation widely recognized as one of its core driving factors (38, 39).

#### 2.2.1 *β*-cell dysfunction and systemic inflammation in T2DM pathogenesis

The core pathophysiology of T2DM involves insulin resistance and *β*-cell dysfunction, the latter being a key feature of disease occurrence and progression. In the early stages, β cells can compensate for insulin resistance by increasing insulin secretion to maintain normal blood glucose levels. However, as the disease progresses, *β*-cell function gradually declines, ultimately leading to insufficient insulin secretion and hyperglycemia (40). This process involves the interaction of oxidative stress, endoplasmic reticulum stress, and chronic inflammation, further accelerating β-cell damage (39).

Systemic inflammation plays a critical role in T2DM, regarded as a state of “metabolic inflammation.” Inflammatory markers such as interleukin 6 (IL-6), interleukin 1β (IL-1β), C-reactive protein (CRP), and tumor necrosis factor *α* (TNF-α) are closely associated with the occurrence and development of T2DM (41, 42). Studies have found that levels of IL-1*β*, IL-18, and TNF-α are elevated in T2DM patients, and vitamin D intervention can partially reduce their levels (43). These inflammatory factors also exacerbate dysfunction by activating inflammatory pathways such as Toll-like receptor 4 (TLR4) and nuclear factor κB (NFκB) in pancreatic β cells (44). Additionally, obesity is closely linked to insulin resistance, and visceral adipose tissue is a major source of early inflammation. Platelets also play an important role in this process, exacerbating systemic inflammation and cardiovascular risks associated with T2DM by releasing inflammatory molecules and participating in coagulation (45).

There is a complex interaction between *β*-cell dysfunction and systemic inflammation. Inflammation directly damages *β* cells and exacerbates insulin resistance, forming a vicious cycle that accelerates T2DM progression (38). Studies have shown that PAK family kinases (PAK16) play a role in β-cell dysfunction, obesity, and insulin resistance, serving as potential molecular targets (46). In summary, the interaction between β-cell dysfunction and chronic inflammation constitutes the core pathological link in T2DM pathogenesis.

#### 2.2.2 Application of AI in simulating and revealing T2DM pathogenesis

AI and ML technologies are being widely applied in T2DM diagnosis, prediction, treatment management, and mechanism research.

In prediction and early diagnosis, AI utilizes EHRs and biomarker data to achieve early disease prediction and risk stratification, using ML techniques to assess the predictive value of inflammatory biomarkers in T2DM development (41). Comprehensive inflammatory indicators including neutrophil-to-lymphocyte ratio (NLR), monocyte-to-lymphocyte ratio (MLR), Systemic Inflammatory Response Index (SIRI), and Systemic Immune-Inflammation Index (SII) have prognostic value for predicting mortality risk in patients with diabetes and prediabetes (47). AI-driven digital biomarkers are also revolutionizing T2DM screening, complication diagnosis, and management.

In mechanism simulation and insight, AI can process multi-omics data such as genomics, transcriptomics, and proteomics to reveal the complex molecular mechanisms of T2DM. Boolean network models simulate *β*-cell apoptosis and insulin resistance mechanisms, clarifying the triggering roles of endoplasmic reticulum stress, oxidative stress, and cytokines (48). AI and ML technologies can also identify brain function changes associated with T2DM-related cognitive impairment through resting-state fMRI data analysis (49). Additionally, preliminary achievements have been made in AI research on angiogenesis and inflammatory mechanisms, helping to understand the role of these complex interactions in the disease.

In drug discovery and treatment optimization, AI can be used to identify new drug targets or repurpose existing drugs to improve T2DM treatment outcomes. Studies have found that 1,25(OH)2D3 can inhibit pancreatic stellate cell activation and promote insulin secretion, suggesting a potential therapeutic strategy. It also demonstrates histological and ultrastructural changes in the T2DM state, as well as the impact of inflammation on body weight, food intake, and water intake, providing a basis for AI to simulate and predict the effects of therapeutic interventions (43). Furthermore, AI helps optimize existing treatment strategies such as insulin therapy to delay *β*-cell failure (50) and can assist in analyzing the mechanism of action of complex drugs in T2DM treatment. For example, the traditional Chinese medicine compound Puren Dan (PRD) improves T2DM symptoms by regulating gut microbiota and their metabolites (51). This study compared differences in cell membranes, lipid rafts, pancreatic *β*-cell function, insulin secretion, blood glucose, inflammation, and lipid metabolism between T2DM and PRD-regulated states, providing visual information for AI analysis of drug mechanisms.

Additionally, oxidative stress is a key factor in T2DM progression (52). The Sirtuin family, including Sirt1, Sirt3, and Sirt6, are key regulators of oxidative stress, and their dysfunction is closely associated with T2DM and its complications (52). Studies have found that AI can analyze their protein interaction networks and regulatory mechanisms in T2DM. This research describes the role of Sirtuins in glucose homeostasis, *β*-cell redox balance, and alleviation of oxidative stress damage, as well as their relationship with T2DM-related complications (such as retinopathy, cardiovascular disease, and liver fibrosis), providing a basis for constructing the oxidative stress mechanism of T2DM.

In summary, significant progress has been made in AI research on T2DM, but challenges remain. In terms of data requirements and model interpretability, higher-quality multi-omics and longitudinal datasets are needed to improve model interpretability to support clinical decision-making. In terms of multi-level integration and personalized application, AI models that can integrate information at the molecular, cellular, tissue, and organ levels are required to more comprehensively simulate the dynamic processes of *β*-cell dysfunction and systemic inflammation, optimizing personalized assessment and treatment plans. Additionally, in emerging research directions such as the association between wearable devices and neurodegenerative diseases, real-time monitoring of blood glucose and inflammatory indicators through wearable devices should be combined with digital interventions (such as personalized dietary and exercise recommendations) to improve patient management and explore the association between T2DM and neurodegenerative diseases.

### 2.3 AI-based integration of multi-omics data

With the development of high-throughput omics technologies (genomics, metabolomics, and microbiomics) and AI algorithms, integrating multi-omics data to accurately predict T2DM pathogenesis has become a research frontier (53).

#### 2.3.1 Application of multi-omics data in predicting T2DM pathogenesis

Genomics primarily studies the association between individual genetic variations such as SNPs and T2DM susceptibility (54). Through Genome-Wide Association Studies (GWAS), researchers can identify gene loci associated with T2DM risk. However, single genomic data cannot fully explain the complexity of T2DM, requiring combined analysis with metabolomics, microbiomics, and other data.

Metabolomics studies the overall changes of small-molecule metabolites, reflecting physiological and pathological states. T2DM patients exhibit disorders in metabolism such as blood glucose, insulin, lipids, and amino acids (55). These metabolites can serve as potential biomarkers for early diagnosis and prediction, such as using targeted quantitative metabolomics data combined with ML to predict the 4-year incidence risk of T2DM (56).

In the field of microbiomics, the gut microbiota is considered an important environmental factor influencing T2DM pathogenesis. Dysbiosis in the composition and function of the microbiota has been confirmed by relevant studies to be associated with core pathophysiological processes of T2DM such as insulin resistance, inflammatory responses, and abnormal energy metabolism (57). For example, the gut microbiota metabolite imidazole propionate affects insulin signaling and key metabolic processes (58). The subgingival microbiota of patients with periodontitis differs from that of non-diabetic individuals, suggesting that oral microbiota may be involved in the pathological process of T2DM (59).

#### 2.3.2 Methodological insights into AI integration of multi-omics data

AI excels at addressing the high-dimensional and heterogeneous challenges of massive multi-omics data, achieving data fusion through advanced algorithms. For example, dimensionality reduction techniques [e.g., principal component analysis (PCA) and t-distributed Stochastic Neighbor Embedding (tSNE)] are used to reduce data noise, or feature selection algorithms (e.g., LASSO and RF) are employed to screen key biomarkers (56, 60). This helps extract meaningful patterns and potential biological connections from complex data.

Currently, various ML algorithms have been applied in T2DM prediction and mechanism research. Tree-based boosting approaches such as gradient boosting decision trees (GBDT) and XGBoost are suitable for non-linear relationships and high-dimensional data. A study found that integrating metabolomics data can improve T2DM prediction accuracy (60). Traditional ML models such as SVM and LR have advantages in T2DM risk classification when the data volume is relatively small or features are clear (56). Faced with complex biological networks and massive data, DL models can automatically learn complex patterns and hierarchical features in data, thereby discovering potential associations that are difficult to capture by traditional methods, such as predicting diabetes complications through analyzing image data (such as retinal images) (61). Additionally, semi-supervised learning and unsupervised learning can be used to mine hidden structures in data to assist in patient subtype classification.

#### 2.3.3 Pathogenesis and future prediction from a comprehensive perspective

AI integration of multi-omics data can construct a more comprehensive map of T2DM pathogenesis and achieve more accurate predictions. As summarized in Table 2, this module covers the application value of genomics, metabolomics, and microbiomics, and their integration via AI algorithms to address high-dimensional data challenges. It reveals that T2DM pathogenesis is not the result of a single factor but a complex network of multi-level and multi-system interactions [for example, genetic variations affect gut microbiota, which in turn alter host metabolites leading to insulin resistance and T2DM occurrence (62)]. It captures “cross-omics” associations such as gene–microbe–metabolite interactions that are difficult to discover through traditional research. For example, METTL3 affects *β*-cell function and insulin secretion and plays a role in complications caused by T2DM (63).

**Figure 1:**
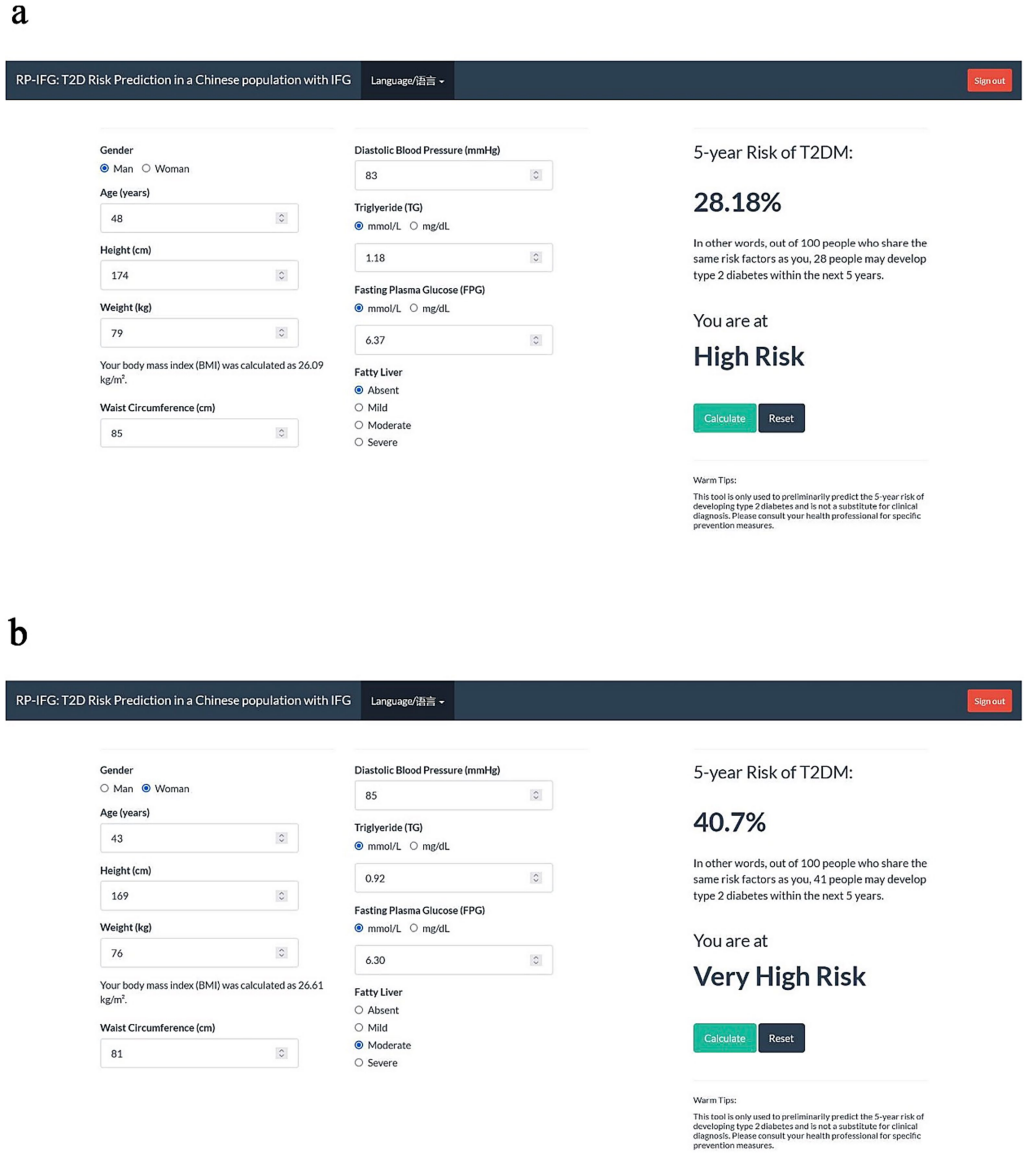
Clinical examples. Using RPM-IFG system to calculate 5-year risk of type 2 diabetes for a man **(a)** and a woman **(b)** with IFG. Figure adapted with permission from Zheng et al. (64).

In terms of early prediction, AI-driven multi-omics prediction models can identify high-risk individuals before the onset of clinical symptoms. As shown in Figure 1, scholars such as Manqi Zheng developed and validated a model for predicting the 5-year T2DM risk based on clinical data and a more effective T2DM risk prediction tool for Chinese populations with Impaired Fasting Glucose (IFG) (64). Combining CGM data with AI can optimize blood glucose management and risk stratification, reflecting the clinical potential of combining omics data with real-time monitoring data. Additionally, in terms of personalized treatment, AI integration of multi-omics data can achieve precision medicine by predicting patients’ responses to specific drugs [e.g., sodium–glucose cotransporter 2 (SGLT2) inhibitors] or recommending specific lifestyle interventions based on their unique biological characteristics (65), aligning with the development trend of multi-omics in endocrinology.

AI integration of multi-omics data to predict T2DM pathogenesis still faces numerous challenges. First, in terms of data standardization, data formats generated by different platforms and laboratories are inconsistent, and there are batch effects, requiring more effective standardization and integration methods (54). Second, in terms of model interpretability, although AI can discover associations, revealing their underlying causal relationships remains challenging, which urgently requires verification through biological experiments (60). Additionally, in terms of large cohort studies, obtaining large-scale, multi-center, long-term follow-up cohorts is crucial to capture the dynamic changes of T2DM progression [e.g., a study on the prospective transition of A1c levels after gestational diabetes mellitus (GDM) used polymorphic Markov models to analyze follow-up data for up to 9 years, suggesting the potential of AI methods in simulating long-term disease progression (66)]. Finally, in terms of clinical translation and ethics, how to effectively translate research results into clinical practice and address ethical issues such as data privacy, fairness, and accessibility are also key research directions (67).

Importantly, the mechanistic insights into T2DM pathogenesis delineated by AI—encompassing the decipherment of G × E interactions, simulation of β-cell dysfunction and inflammatory pathways, and integration of multi-omics data—are not confined to theoretical frameworks; rather, they establish a direct translational pipeline from biological discovery to personalized clinical practice, thereby laying the foundation for targeted individual-level care. This translational value directly addresses the challenge of translating research outcomes into clinical applications. Concrete instances of such translation include: (1) AI-mediated identification of the ABCA1 69C > T polymorphism as a key biomarker associated with T2DM susceptibility in rural Han Chinese populations (26)—a finding that has been translated into clinical practice, wherein carriers of this variant are stratified as high-risk individuals via AI-driven risk assessment tools. This stratification further triggers a tailored remote monitoring protocol, incorporating weekly CGM data analysis and monthly dietary guidance delivered through mHealth applications (e.g., recommendations for reduced refined carbohydrate intake) to mitigate inherent genetic predisposition (19); (2) AI-enabled revelation that the gut microbiota-derived metabolite imidazole propionate acts as a driver of insulin resistance (58)—this mechanistic insight has been leveraged to inform medication selection, with AI systems recommending SGLT2 inhibitors for patients with elevated imidazole propionate levels (detected via metabolomic profiling). This recommendation is supported by evidence that SGLT2 inhibitors counteract the deleterious effects of this metabolite on glucose metabolism (65). These examples collectively illustrate how AI bridges mechanistic understanding and clinical implementation, facilitating the transition from universal, “one-size-fits-all” T2DM management to precision care strategies tailored to the unique biological and environmental profiles of individual patients—an integration further elaborated in the following section on individual-level T2DM care.

## 3 Application of AI-enabled digital health tools in individual-level T2DM care

Digital health tools are gradually becoming important components of patient management and medical services in T2DM. They promote a shift from episodic clinic-based care to continuous monitoring and personalized intervention, thereby reshaping both patient self-management and healthcare delivery. In this section, we synthesize the evidence along a monitoring-to-intervention pathway and treat mobile applications and wearable devices primarily as implementation modalities that operationalize remote monitoring and deliver just-in-time support.

### 3.1 Remote monitoring and personalized interventions in T2DM management

#### 3.1.1 Implementation of remote monitoring

AI integrates multi-source data to achieve continuous and seamless remote monitoring of T2DM patients. CGM data undergo AI time-series analysis to identify blood glucose fluctuation patterns and support individualized dietary and behavioral feedback. For example, AI can help patients understand the association between postprandial blood glucose peaks (e.g., exceeding 180 mg/dL 2 h after meals) and lifestyle factors, enabling more targeted self-management (68). In addition, CGM data can also be used to stratify T2DM patients, prioritizing those based on disease severity or immediate intervention needs, which is relevant to both clinical follow-up and population management (23).

Novel hybrid multimodal wearable sensors collect various physiological signals beyond blood glucose (e.g., metabolites such as electrolytes, stress, and hydration status) and electrocardiograms (such as heart rate of 98 bpm). Combined with AI-based regression, classification, and clustering, these sensors support personalized and potentially non-invasive monitoring of biomarkers and can assist in identifying metabolic risk patterns (69). Telemedicine platforms combined with AI further extend monitoring capacity by enabling data-driven follow-up and adherence support. For example, the MobiDiabet system is a mobile remote monitoring platform designed for T2DM patients, providing structured follow-up and adherence facilitation; preliminary usability evaluations indicate an overall satisfactory usability level despite certain limitations (70). Additionally, AI-driven digital biomarkers provide decision support to healthcare professionals through wearable-derived data (e.g., heart rate, sleep patterns, and activity) and medical records.

Collectively, these monitoring systems rely on scalable implementation modalities—most notably AI-enabled mobile applications and wearable devices—which enable real-time sensing, feedback delivery, and long-term engagement.

#### 3.1.2 Provision of personalized treatment recommendations

**Figure 2:**
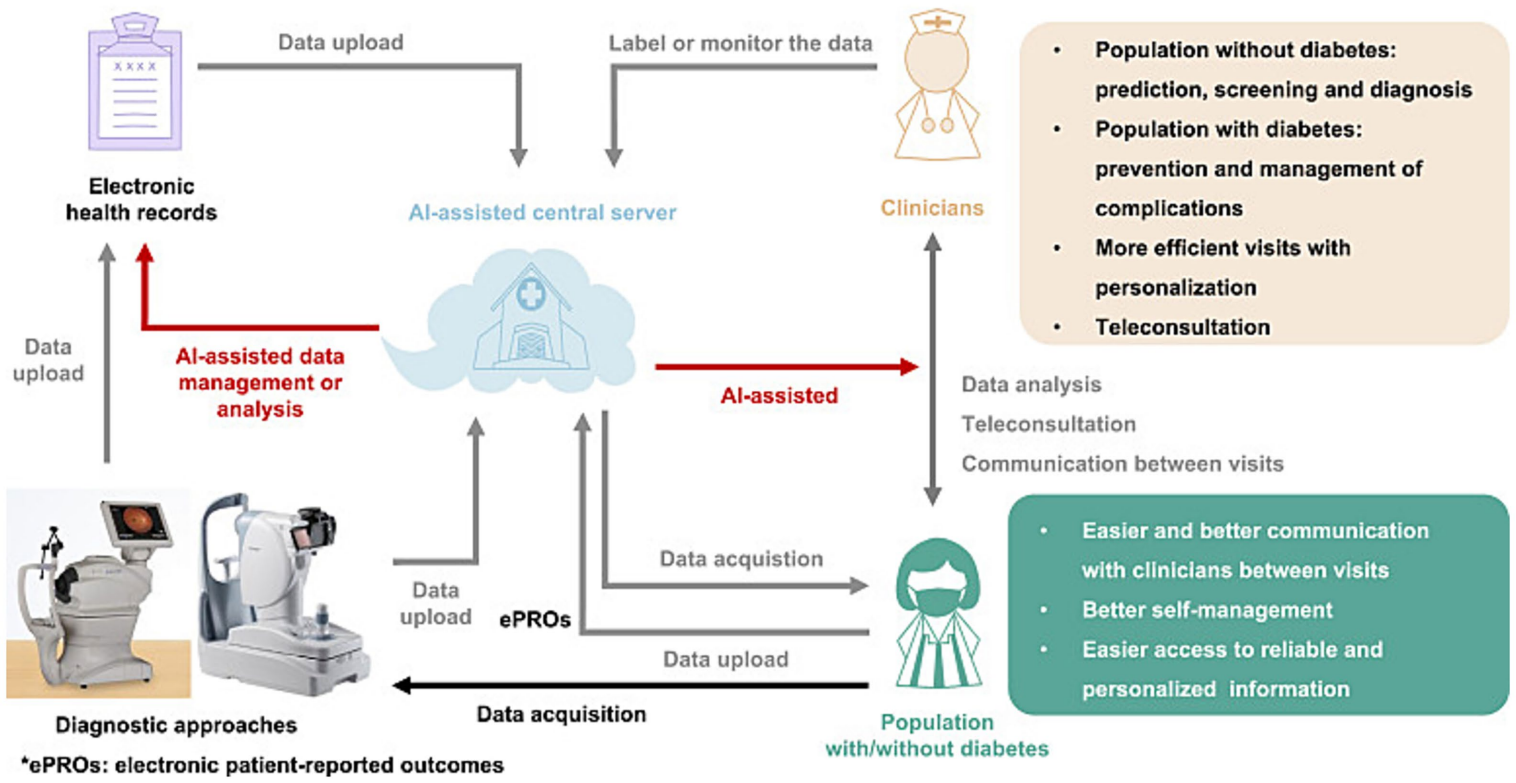
Proposed AI-assisted digital healthcare ecosystem for diabetes management. Figure adapted with permission from Guan et al. (74).

AI provides personalized recommendations for T2DM patients through multimodal data analysis. DL integrates genomic information, environmental factors, behavioral factors, social factors, imaging data, and medical records to identify disease risk factors and generate customized medical and lifestyle recommendations (71, 72). This supports coordinated care by linking patient data with individualized treatment plans. In clinical decision support systems, AI can analyze massive medical data (including patient records, research literature, and clinical guidelines) to provide evidence-based insights for clinical decision-making (73). For instance, as shown in Figure 2, an AI-assisted central server constitutes the core of a proposed digital healthcare ecosystem for diabetes management: It receives data from EHRs and diagnostic methods, manages, and analyzes it through AI, predicts diabetes risk in non-diabetic populations, and supports complication prevention in diabetic patients (74).

Driven by AI, digital twins—a virtual replica of an individual’s physiological system, constructed by fusing real-time CGM data, EHRs, genetic information, and lifestyle records to simulate disease progression and treatment responses dynamically—have attracted attention as a potential approach to integrate multi-dimensional datasets and simulate disease trajectories, offering opportunities for personalized treatment and potentially improved long-term management (75). A practical validation of this technology is the “DiaTwin” system developed by the University of Toronto: tested in 250 T2DM patients with comorbid kidney disease, the system simulated the impacts of SGLT2 inhibitor dosage adjustments and sodium restriction on blood glucose and renal function. By enabling clinicians to test “what-if” scenarios (e.g., “How would glucose change if the patient reduces carbohydrate intake by 30g?”) before implementing real-world interventions, it reduced acute kidney injury episodes by 38% and improved HbA1c control (<7.0%) by 45% compared to standard care (75).

Beyond digital twins, patient participation and self-management remain essential in chronic disease control, and AI tools can enhance engagement through medication reminders, blood glucose monitoring prompts, lifestyle coaching, and tailored educational resources. For example, the AI-assisted Health Education Accurately Linking System (AIHEALS) provides personalized and precise management for T2DM patients (76). In prediction models and treatment response prediction, AI algorithms can analyze historical patient data, genetic markers, and other relevant factors to predict disease progression and responses to different treatment plans, enabling more proactive and individualized strategies (73). AI has also been explored in optimizing drug delivery systems to achieve more precise dose control.

### 3.2 Mobile applications and wearable devices as implementation modalities

AI-driven mobile applications and wearable devices combine multimodal sensors with AI algorithms to enable real-time monitoring, deliver reminders, and support lifestyle interventions. Rather than representing a separate application layer, these technologies function as delivery channels that operationalize remote monitoring and personalized support at scale (77).

#### 3.2.1 Real-time blood glucose monitoring

Real-time blood glucose monitoring is a core use case of AI-enabled wearables and mobile applications. Compared with traditional finger-prick monitoring, AI has been combined with Photoplethysmography (PPG) and Internet of Things (IoT)-enabled mobile integration to explore non-invasive monitoring methods (78). PPG is a non-invasive optical technique that detects blood volume changes, while IoT connects wearable devices to mobile platforms for real-time data transmission. AI-enhanced CGM systems can analyze glucose data in real time, identify patterns, and send timely alerts to patients and providers to enable intervention (e.g., when glucose approaches dangerous thresholds) (73). Some IoT devices combine CGM with attention-based RNNs to support 24-h glucose prediction, facilitating proactive intervention (79). AI models have also been developed to leverage multivariate glucose data for improved spatiotemporal modeling beyond univariate approaches.

The Dexcom G7 CGM with AI prediction was prospectively validated in 1,024 T2DM patients, reducing severe hypoglycemia episodes by 40% over 6 months compared with finger-prick monitoring (23). RCTs show AI-driven CGM increases TIR by 15–20% (target: >70%), reduces HbA1c by 0.6–0.8%, and decreases emergency admissions for hyperglycemia by 25% (69). These systems have been scaled in high-income settings via reimbursement mechanisms, while coverage remains limited in LMICs due to device costs ($150–$500/device) (80). A non-invasive AI-PPG device failed a prospective trial (*n* = 300) due to poor accuracy (mean absolute relative difference = 18% vs. CGM), with no meaningful impact on TIR. In LMIC settings, motion artifacts and context-specific noise can lead to high false-alarm rates (up to 30%), reducing adherence and trust (81).

Cost-effectiveness is highly context-dependent. In Germany, a 5-year RCT (*n* = 2,000) indicated that AI-driven CGM (cost: €300/year/patient) reduced complications by 28% and saved €1,200/patient/year in long-term costs—cost-effectiveness ratio (CER) of €0.25 per quality-adjusted life year (QALY) gained (66). This aligns with Digitale Gesundheitsanwendungen (DiGA) reimbursement criteria (CER < €30,000/QALY). In rural Kenya, the low-cost MobiDiabet Lite (€20–30/device) achieved a CER of €1.8/QALY by reducing emergency admissions by 25% (saving €80/patient/year) (70). Conversely, a non-invasive AI-PPG monitor in India showed negative cost-effectiveness due to insufficient accuracy and no complication reduction (CER > €50/QALY) (78).

However, LMICs face significant implementation challenges due to foundational technical gaps (section 5.1), including device affordability ($150–$500/device) and connectivity issues (80). LMICs-adapted models therefore emphasize low-cost transmission [e.g., short message service (SMS)], lightweight algorithms for low-power devices, and context-specific model training to mitigate overfitting and false alarms (79).

#### 3.2.2 Medication reminders

AI-driven mobile applications can improve patients’ medication adherence through personalized reminder schedules and multi-channel prompting. Integration with IoT devices such as smart pillboxes and radio frequency identification (RFID)-based tracking enables basic adherence logging and feedback to care teams (82). Reminder modalities may include in-app notifications, SMS, phone calls, visual/auditory cues, and caregiver alerts (83). Digital twins of smart devices have also been explored for test automation and reliability assurance of dispensers and monitors. As shown in Table 1, digital twins of T2DM patients integrating CGM and EHR data showed potential for predicting hypoglycemia events in a small RCT (*n* = 150), but scalability remains limited due to computational demands and data completeness constraints in complex older adult patients (75).

In LMICs, literacy constraints and device limitations can make text-heavy apps unusable, and on-device computation for complex medication interaction algorithms may exceed available smartphone resources (84, 85). Context-adapted approaches therefore emphasize voice-based reminders in local languages and community health workers (CHWs)-supported workflows. For example, the Kenyan app “Diabetes Care Africa” delivered audio reminders and shared adherence signals via SMS with CHWs, improving adherence and associated outcomes in prospective validation (86, 87). Low-cost RFID-enabled pillboxes ($5–$10) also provide feasible adherence tracking without requiring high-end smartphones (82). Cost-effectiveness evidence further suggests that appropriately adapted interventions can be economically attractive within public health systems, whereas non-adapted text-only solutions may underperform where literacy barriers persist.

#### 3.2.3 Lifestyle interventions

Future directions include wearable bioelectronics, predictive analytics, and edge AI (a lightweight AI paradigm). It deploys simplified algorithms directly on local devices (e.g., smartphones and low-cost wearables), enabling real-time data processing without relying on cloud servers or stable internet. This feature makes edge AI particularly critical for resource-limited settings to enable local processing and reduce latency (88–90). Nevertheless, these advances must be evaluated in relation to data quality, interoperability, patient acceptance, and implementation feasibility to ensure real-world benefit.

AI-driven wearables and mobile applications support lifestyle interventions by tracking activity, sleep, and physiological states and providing personalized feedback. Because lifestyle modification depends on sustained engagement, digital interventions increasingly incorporate Behavior Change Techniques (BCTs), with AI/ML used to tailor prompts and adapt coaching intensity over time (91). Mobile self-monitoring tools have been designed to strengthen self-management skills and may support the prevention of complications, showing promising usability outcomes in small cohorts (92). In addition, AI-supported risk assessment models integrating EHRs and multi-omics data may help identify early signs of deterioration and facilitate timely intervention (93). Digital health interventions (DHIs)—including SMS, smartphone apps, and web platforms—have shown measurable effects on glycemic outcomes, though effectiveness is mediated by usability, context adaptation, and sustained adherence (91).

### 3.3 Intelligent integration of medication and lifestyle interventions

To optimize outcomes and reduce public health burden, recent research increasingly emphasizes integrating medication treatment with lifestyle interventions through AI (50). Conceptually, this represents a synthesis layer that builds upon monitoring and intervention modalities, aiming to jointly optimize pharmacological strategies and behavioral support over time.

AI models analyze behavioral and physiological data to tailor medication plans and lifestyle guidance, enabling finer adjustment of dosing and intervention intensity. For instance, in complex comorbidity contexts, AI can assist in optimizing initiation strategies for drugs such as glucagon-like peptide-1 receptor agonists (GLP-1 RAs) and SGLT2 inhibitors (94). AI can also predict adherence risks by identifying behavioral and socioeconomic drivers of treatment failure, supporting targeted support strategies (95). Interdisciplinary care models—such as AI-assisted glucose management systems involving clinical pharmacists—illustrate how integrated workflows can improve care quality through coordinated medication adjustment and monitoring (96). Large initiatives such as the AI-ECG Risk Estimator-Diabetes Mellitus (AIREADI) highlight the broader goal of leveraging AI to study resilience and enable holistic long-term T2DM management (97).

From a public health perspective, integrated approaches can reduce long-term costs and improve quality of life, although the magnitude of benefit varies across settings and depends on feasibility and affordability (75). Advanced approaches (e.g., digital twins) may demonstrate cost-effectiveness in high-resource settings but remain challenging to scale in low-resource environments due to infrastructure and budget constraints (75). Overall, the integration of medication optimization with personalized lifestyle support represents a promising direction, but its scalable impact is contingent on implementation context.

### 3.4 AI optimization of resource allocation and treatment adherence

AI has advantages in improving adherence and optimizing healthcare resource allocation, which is critical given the high prevalence and global burden of T2DM (98). AI-driven tools can identify individuals at risk of poor adherence by integrating multi-source data, enabling proactive interventions and more efficient follow-up (74). RL models have been explored for personalized multimorbidity management plans and have shown prospective validation in specific patient cohorts, improving glycemic and cardiovascular outcomes without increasing hypoglycemia risk (95, 99). AI also supports public health management by predicting long-term T2DM risk using EHR features and enabling earlier intervention strategies (100).

At a system level, AI-driven analysis of EHRs can support resource planning (e.g., workforce scheduling, equipment utilization, and supply chain management), especially in resource-constrained settings (101). Nevertheless, real-world implementation remains limited by fragmented data, interoperability challenges, and the need to improve clinician trust and patient acceptance—issues that are examined in subsequent sections on technical/ethical constraints and governance frameworks (102).

## 4 System-level applications of AI in public health T2DM management

As AI-enabled digital health tools are deployed at scale, their impact extends beyond individual clinical outcomes to system-level performance, equity, and governance. Against this backdrop of their popularization, the management of T2DM has evolved from traditional clinical interventions to more comprehensive approaches that rely heavily on digital health technologies like CGM, digital biomarkers, and telemedicine—a shift that aligns with the exploration of AI’s system-level applications in population-level public health management of T2DM. Notably, the large-scale application of these AI and digital health tools in T2DM public health management is underpinned by technical infrastructure, while core challenges that are cross-cutting and foundational persist. These challenges affect individual-level care, population health interventions, and equity outcomes; they act as the root cause of many systemic issues (e.g., digital divide and uneven access) and are elaborated below to clarify their universal impact.

### 4.1 Foundational technical challenges

#### 4.1.1 Compute, infrastructure, and maintenance costs

High computational costs and inadequate infrastructure represent primary bottlenecks to AI scalability in T2DM management, particularly in LMICs. Healthcare budgets in LMICs are only 10–20% of those in HICs on a per capita basis, with annual per capita healthcare spending often below $100 (103).

Advanced AI models, especially DL architectures designed for multi-omics data integration (a critical capability for personalized T2DM risk stratification and management), require substantial hardware resources: specifically, 16–32 GB of graphics processing unit (GPU) memory. This requirement is prohibitive in LMICs, where 95% of hospitals lack access to such GPU infrastructure (35). Even DL models for multi-omics integration that have demonstrated prospective validity in HICs (e.g., achieving an AUC of 0.83) have failed to scale in LMICs due to this hardware deficit (35). Infrastructure limitations have also extended to energy and connectivity. The energy consumption of AI infrastructure is unfeasible in resource-constrained regions: A single DL model training session emits 2.5 tons of carbon dioxide (CO₂), equivalent to the annual emissions of 500 rural households in Kenya (104). For LMICs already facing chronic energy shortages, this carbon footprint is both economically unaffordable and environmentally unsustainable. Unstable internet connectivity further exacerbates implementation challenges; for example, a Federated Learning (FL) pilot (a privacy-preserving AI training approach) for T2DM in Nigeria was discontinued after 6 months due to unreliable internet access, resulting in no meaningful impact on clinical outcomes (105).

Beyond initial compute costs, deploying AI tools requires ongoing maintenance, including software updates, server storage, and technical troubleshooting. Annual maintenance costs average $2,000 per clinic—a sum that diverts limited resources from essential T2DM services, such as insulin provision and foot ulcer screening (11), which are already underfunded in LMICs. As highlighted in Table 1, edge AI emerges as a low-resource-adapted solution to address these cost and infrastructure barriers: It has been scaled in rural China (covering over 200,000 patients) for T2DM screening, achieving an AUC of 0.71 with no internet requirement and reducing diagnostic delays by 40% (106). A representative practical application is the “RuralDiab” edge AI tool, deployed in 60 rural clinics in Yunnan, China—its performance and scalability advantages are clearly summarized in Table 1. This tool integrates a lightweight XGBoost variant that analyzes basic clinical data (age, BMI, fasting glucose) and wearable-derived step counts locally, and without internet connectivity, it achieves a higher T2DM screening AUC of 0.76 while generating personalized dietary recommendations (e.g., “Increase bitter melon intake, reduce polished rice”) instantly. Over 2 years, 12,000 high-risk individuals using “RuralDiab” had a 21% lower T2DM incidence than those receiving usual care (106).

#### 4.1.2 Interoperability and workflow integration

Interoperability failures and poor integration with existing healthcare workflows undermine the utility of AI tools for T2DM management, particularly in LMICs. A foundational barrier is the fragmentation or absence of EHRs systems: 70% of LMICs lack cohesive EHR platforms, and those that exist often operate in silos (107). AI tools for T2DM—such as those for real-time glucose trend analysis or risk prediction—rely on seamless access to patient data (e.g., historical glucose readings, medication adherence, and comorbidity records) from EHRs; without this integration, model accuracy and clinical relevance are compromised. Even in settings with partial EHR adoption, interoperability issues persist. Incompatible data formats between AI tools, wearables (e.g., CGM), and EHRs systems create data silos (108). For instance, a wearable device may generate glucose data in a proprietary format that cannot be imported into a clinic’s EHR, preventing AI models from incorporating real-time patient data into T2DM management decisions. This lack of data harmonization reduces the utility of AI tools, as models are unable to leverage comprehensive patient information to refine risk assessments or treatment recommendations.

Additionally, AI tools often fail to align with existing clinical workflows in LMICs. Many tools are designed for high-resource settings with structured data collection processes and dedicated technical staff, rather than the fast-paced, resource-scarce environments of LMIC primary care clinics. This misalignment increases workflow disruption for healthcare providers, who may lack the time or support to adapt their routines to accommodate AI tool use—further reducing adoption and scalability.

#### 4.1.3 Human capacity and operational readiness

A lack of human capacity and operational readiness among healthcare workers in LMICs represents a critical barrier to effective AI implementation for T2DM management. AI tools for T2DM (e.g., those generating risk scores, interpreting imaging for diabetic retinopathy, or optimizing medication regimens) require providers to understand how to operate the tools, interpret outputs, and integrate recommendations into clinical practice. However, healthcare workers in resource-limited settings often lack training in AI literacy and tool-specific operation. Empirical evidence highlights this gap: Only 15% of primary care providers in South Asia report confidence in interpreting AI-generated T2DM risk scores (109). This low confidence stems from limited access to structured AI training programs, which are rarely included in pre-service or in-service medical education curricula in LMICs. Without training, providers may either underutilize AI tools (e.g., ignoring risk score recommendations) or misinterpret outputs (e.g., overestimating T2DM risk based on an incomplete understanding of model limitations), leading to suboptimal patient care.

Operational readiness is further compromised by a shortage of technical support staff. In LMICs, most primary care clinics do not employ dedicated IT personnel to troubleshoot AI tool malfunctions (e.g., software glitches and data import errors) or provide on-demand assistance to providers. This lack of support increases provider frustration and reduces tool adoption, as workers cannot resolve technical issues in a timely manner—particularly critical in settings where providers already face high patient loads and time constraints. Finally, provider attitudes toward AI can influence operational readiness. Some healthcare workers in LMICs express skepticism about AI tools, citing concerns about model transparency (e.g., “black box” DL outputs) or fears that AI may replace human judgment (84). These attitudes, combined with limited training, create a barrier to proactive adoption, even when AI tools are technically accessible and affordable.

In summary, these constraints indicate that technical optimization alone is insufficient for AI-enabled T2DM management—sustainable deployment requires governance mechanisms that operationalize accountability, privacy protection, fairness assurance, and lifecycle oversight. Notably, the foundational technical challenges underpinning these constraints directly shape equity outcomes (section 5.2) and policy responses (section 5.3), forming a critical chain that must be addressed holistically to achieve equitable scale.

### 4.2 Data-intensive digital health infrastructure for population T2DM management

Building on the foundational technical capabilities (section 5.1) that address interoperability, infrastructure, and capacity gaps, data-intensive digital health infrastructure serves as the backbone for population-level T2DM management—integrating real-world data to generate actionable insights across prevention, treatment, and monitoring.

#### 4.2.1 AI-enabled data streams and real-world data ecosystem

AI plays a pivotal role in shaping AI-enabled data streams and optimizing the real-world data ecosystem for T2DM full-cycle management. By integrating diverse real-world data—including EHRs, genomic data, wearables, electronic patient-reported outcomes (ePROs), and CGM—AI generates actionable insights: customized treatment plans, insulin dose adjustments, and early warnings for complications (e.g., kidney disease and neuropathy). AI-driven applications also capture patient adherence and engagement metrics, feeding into data streams to enhance the ecosystem’s granularity and support holistic, proactive care (110).

A key pillar of these data streams is CGM data, whose use is expanding among patients on basal insulin or pharmacologic management (23). Beyond minimizing hypoglycemia and enabling comprehensive glucose control, CGM provides dynamic, patient-generated data that complements traditional sources, enriching the real-world data ecosystem’s diversity. This synergy between AI-enabled streams and integrated real-world data creates a robust infrastructure, empowering accurate predictive modeling and personalized T2DM interventions from prevention to long-term management.

#### 4.2.2 Application scenarios in public health management

AI has significant potential in the full-cycle management of T2DM. First, AI can customize treatment plans and insulin doses for patients based on their medical history, real-time blood glucose levels, and lifestyle. Second, AI can assess and prevent T2DM complications such as kidney disease, neuropathy, and retinopathy and provide customized dietary and lifestyle recommendations by analyzing patient data. Additionally, AI-driven applications can increase patient participation by providing medication reminders and educational resources. Finally, AI can predict disease progression and treatment responses to achieve more proactive and personalized treatment plans (73).

These applications rely on continuous, multi-source data flows, which introduce privacy, security, and governance constraints that must be addressed before scaling—issues further discussed in section 6.

### 4.3 Equity implications of AI-enabled digital health at scale

The foundational technical challenges outlined above (section 5.1) are deeply rooted in equity gaps in digital health for T2DM management: infrastructure deficits, high costs, and capacity limitations—core components of these technical challenges—create uneven access to life-saving digital health tools, leaving marginalized T2DM patients facing disproportionate barriers to access.

#### 4.3.1 Digital divide and access barriers

Marginalized patients with T2DM struggle to fully utilize digital health tools due to unstable internet access, lack of smart devices, and insufficient digital literacy. Such barriers are particularly prominent in LMICs: 70% of patients in sub-Saharan Africa cannot afford wearable devices (priced at $100–$300) (103), 43% of rural areas lack 3G/4G networks for real-time data transmission (109), and 60% of donated wearables in rural India become inoperable within 6 months due to inadequate technical support (84). Gaps in digital literacy further widen this divide: only 28% of older adult T2DM patients in LMICs can independently interpret AI-generated glucose reports, and training programs are scarce due to the limited capacity of healthcare workers (111).

This divide is not limited to cross-country comparisons but also exists within nations. For instance, China’s rural–urban T2DM digital divide is well documented: A national survey (*n* = 50,000 T2DM patients) found that urban patients are 3.2 times more likely to use AI medication reminder apps (45% vs. 14%) and 2.8 times more likely to access telemedicine (38% vs. 14%), leading to a 0.5% higher HbA1c reduction in urban patients over 1 year (112).

While DHIs have been proven effective in supporting T2DM patients’ self-management and improving blood glucose control (113), their real-world effectiveness heavily depends on two key factors: patients’ digital literacy and technical accessibility to digital tools. Specifically, patients in low-income or rural areas lack stable high-speed internet and cannot afford devices; even when equipped with devices, some older adult or low-educated patients struggle to operate complex applications due to insufficient digital skills. This results in low usage rates of digital tools and failure to benefit from AI-assisted tools like the personalized health education system AI Health Education and Precision Linkage System (HEALS) (76, 84).

Notably, the effectiveness of DHIs in addressing these disparities is context-dependent. Although DHIs have been proven effective in HICs, their effectiveness in LMICs depends on context adaptation: A meta-analysis found that DHIs tailored to LMIC barriers (e.g., offline functionality and CHW integration) improve blood glucose control by 0.5–0.8% HbA1c, while unadopted HIC-derived tools show no significant benefit (103). This highlights the need for socioeconomic and cultural factors to be central to DHI design, rather than afterthoughts.

These gaps are not accidental but stem from systemic technical bottlenecks (section 5.1) and socioeconomic determinants.

#### 4.3.2 Structural and socioeconomic determinants shaping uptake and outcomes

Socioeconomic factors have a significant impact on the medical utilization rate and management effect of T2DM patients (84). Factors such as poverty, low educational levels, and insufficient medical resources place marginalized patients at a disadvantage in T2DM management. For example, the medical utilization rate of T2DM patients in slums is seriously insufficient (114). Additionally, cultural differences, language barriers, technology acceptance, and trust in the healthcare system may also affect the effectiveness of AI and digital health tools in different groups (115).

#### 4.3.3 Equity-oriented implementation strategies

To mitigate technology-driven inequities, strategies must target both foundational infrastructure and context adaptation: First, promote affordable technologies and reduce the cost of digital health tools to make them more accessible to marginalized groups. Second, provide digital literacy training for T2DM patients to equip them with the skills needed to use digital health tools. For example, AIHEALS provides personalized and precise management for T2DM patients through AI technology on mobile devices (76). Additionally, provide multi-language and culturally adapted versions of digital health tools. A study by scholar Mihyun Jeong on Korean-American T2DM patients found that sleep quality and physical activity are related to diabetes quality of life, suggesting that cultural and lifestyle differences need to be considered in digital health tools (116).

In addition, scholars such as Seonah Lee proposed in a study that community organizations can be united to promote digital health tools through channels such as community health centers and non-profit organizations and provide on-site support and assistance. Meanwhile, the public–private partnership model in South Korea can be learned from, where the government can cooperate with private medical centers to jointly operate web-based management systems, providing services for hypertension and diabetes patients, including waiving outpatient fees and drug costs, to improve medical accessibility (112).

### 4.4 Policy and health system enablers for scaling AI-enabled T2DM management

Countries are promoting the application of AI in T2DM management through policies, that focus on supporting telemedicine and digital health to address the growing global medical costs, shortages of medical labor, and the increasing challenge of chronic diseases. The integration of AI and telemedicine is considered a key means to improve medical accessibility, efficiency, and diagnostic accuracy.

#### 4.4.1 Reimbursement and HTA as scaling mechanisms

Technical barriers (e.g., high device costs and poor connectivity) limit AI tool adoption in LMICs, making context-adapted reimbursement policies critical (section 5.1).

In terms of health monitoring, the integration of AI into CGM devices has become a key policy focus. Specifically, policies target T2DM’s real-world management pain points—some countries are exploring the application of CGM data combined with HbA1c in T2DM patient triage, aiming to optimize the efficiency of patient risk stratification and intervention priority setting for individuals with poor glucose control (23). The reimbursement mechanism for digital health technologies such as CGM is a key part of policy promotion. The German “Prescription App” (e.g., DiGA) model, as a mature regulatory and reimbursement paradigm, has significant promotional value for the reimbursement of CGM and similar AI-integrated technologies (117). This model not only clarifies the access standards for digital health tools but also establishes a sustainable economic support mechanism for their clinical application.

In terms of digital therapy access, the access and reimbursement mechanisms for digital therapy, as a form of DHIs, are at the core of policy support. For example, the DiGA model allows certified digital health applications to be reimbursed, providing important policy guarantees for the widespread application of digital therapy in T2DM management (117). This model helps promote the transformation of digital therapy from “self-payment” to “medical insurance coverage,” expanding its application scope and accessibility for patients across different socioeconomic groups.

Reimbursement mechanisms, as a key driver for the large-scale promotion of AI tools, consider cost-effectiveness as a core eligibility criterion in policy design. The DiGA model, for instance, has clear requirements: AI tools applying for reimbursement must demonstrate both clinical benefits (e.g., achieving a reduction of ≥0.3% in HbA1c levels) and cost-effectiveness (CER < €30,000/QALY). To date, 12 AI-driven diabetes tools (including CGM analytics and medication reminders) have been approved, covering approximately 1.2 million patients—reducing national diabetes-related healthcare spending by 3.5% (equivalent to €450 million/year) (117). In the US, Medicare has also incorporated AI-enhanced CGM into the reimbursement scope for T2DM patients receiving insulin therapy, with a coverage rate of 85%. This decision is supported by evidence from RCTs, which confirmed that AI-enhanced CGM can reduce the incidence of diabetes complications by 28% and achieve a CER of $1,200 per QALY, meeting the cost-effectiveness standards of public health expenditure (66).

However, there are significant gaps in LMICs. Only 5% of LMICs have incorporated cost-effectiveness into AI reimbursement. For example, India’s Ayushman Bharat scheme excludes AI tools due to a lack of local cost data, despite evidence that low-cost edge AI (with an annual cost of approximately €10 per patient) could save €40/patient/year in long-term diabetes management costs, reflecting the mismatch between policy design and practical evidence in LMICs (106). Such gaps not only limit the popularization of AI tools in resource-constrained regions but also may exacerbate global disparities in T2DM management.

#### 4.4.2 Telemedicine and AI as a public health delivery model

**Figure 3:**
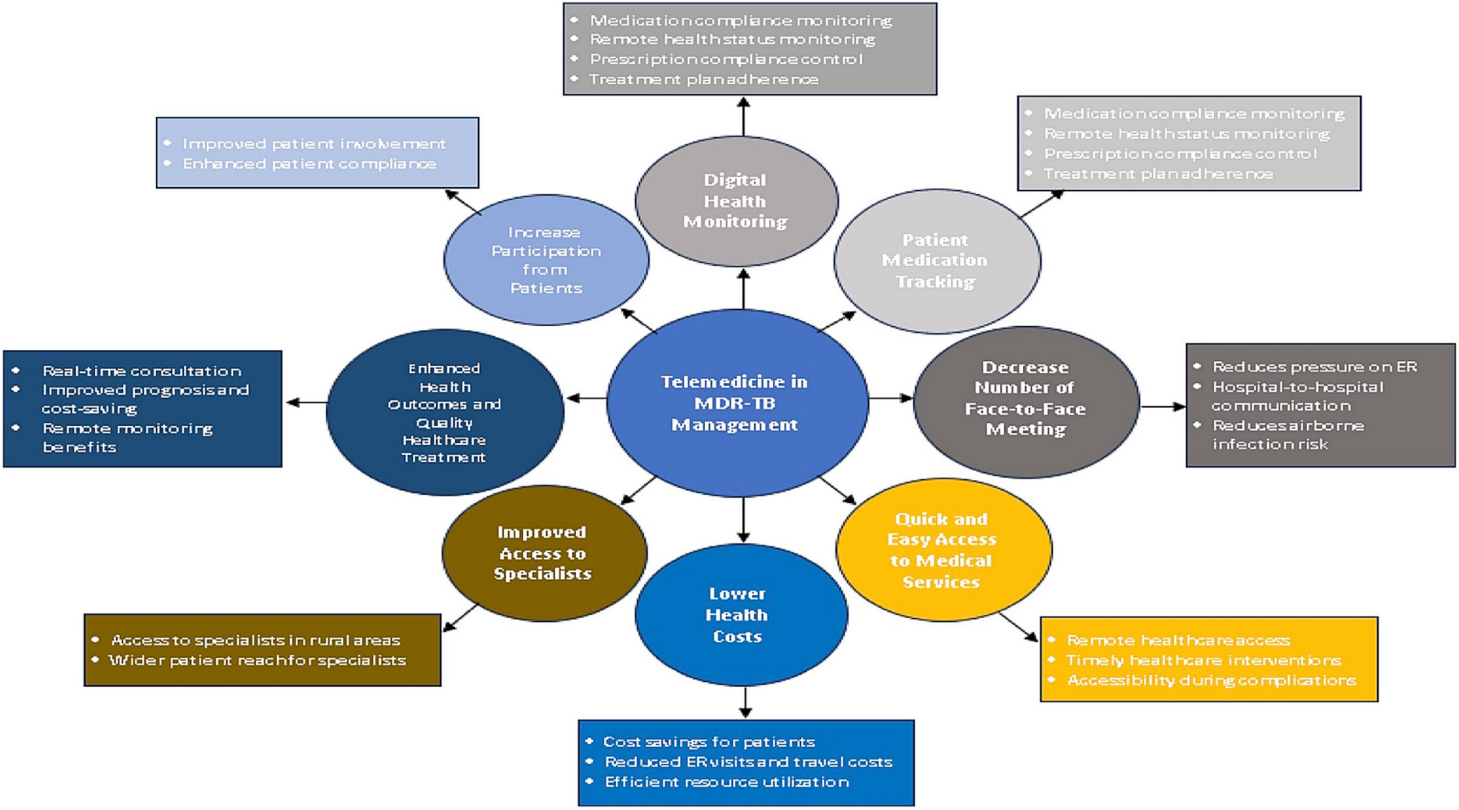
Highlights of roles of telemedicine in preventing MDR-TB. Figure adapted with permission from Olowoyo et al. (118).

Countries are promoting telemedicine and digital health policies in primary healthcare and chronic disease management to address T2DM follow-up and adherence issues. As shown in Figure 3, scholars like Kikelomo S. Olowoyo note telemedicine’s key advantages in chronic disease management, which are equally applicable to T2DM care: digital health monitoring, reduced in-person visits, convenient care access, medical cost reduction, expanded expert access, improved care quality, and enhanced patient participation (118). These advantages are particularly critical for overcoming resource shortages in LMICs.

Notably, telemedicine combined with AI achieves economies of scale in public health, delivering both clinical and economic benefits. A multi-center study in Brazil (*n* = 5,000) demonstrated that AI-driven teleconsultations (costing €15 per visit) reduced in-person T2DM follow-up visits by 40%, translating to €80 in annual savings per patient (from reduced travel and healthcare expenses). For public health systems, this generated €2 million in annual savings for a target population of 50,000 (119). Long-term value is further evidenced by a Swedish initiative, where AI-optimized resource allocation for 100,000 T2DM patients—with an annual maintenance cost of €200,000—reduced complication rates (e.g., diabetic nephropathy and cardiovascular events) by 30%, resulting in €3 million in annual savings from avoided hospitalizations and disability-related costs (98). Countries are actively exploring practical pathways to integrate AI and telemedicine for T2DM management; for instance, the United Arab Emirates (UAE) has integrated AI and telemedicine into its healthcare system to enhance service accessibility, convenience, and doctor–patient communication (120).

**Figure 4:**
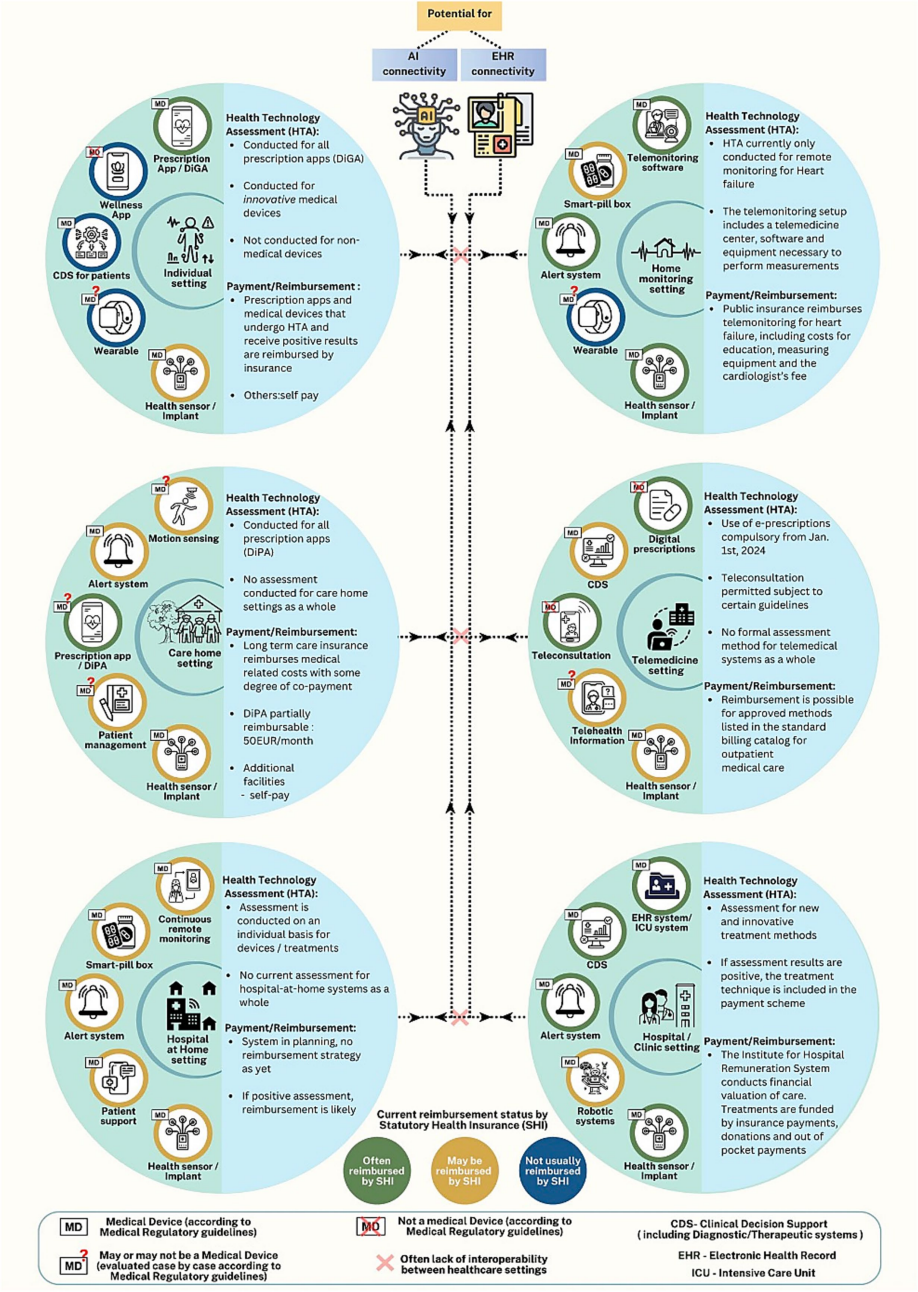
Overview of modular DHT use across different healthcare settings. Figure adapted with permission from Mathias et al. (117).

These data highlight the role of policy-supported telemedicine–AI integration in improving the sustainability of public health systems, particularly in contexts with limited resources. However, DHI gaps persist in LMICs—with applications concentrated in high-income countries/hospitals (103)—requiring region-specific policies to strengthen infrastructure, data sharing, and privacy protection for wider benefit extension. To advance such integration, as shown in Figure 4, scholars such as Rebecca Mathias present an overview of modular digital health tool applications across different healthcare settings, emphasizing that AI connectivity and EHR connectivity are core to unifying these diverse medical technology applications. This indicates policymakers are increasingly focusing on technical solutions to achieve seamless data sharing and functional enhancement within healthcare systems. Additionally, they stress the criticality of Health Technology Assessment (HTA), clear regulatory status, and stable payment/reimbursement mechanisms for the effective adoption of digital health technologies in T2DM management (117).

#### 4.4.3 Summary: what enables scale, and what remains unresolved?

The scaling of AI-enabled T2DM management in public health is underpinned by three interconnected enablers. First, targeted policy and reimbursement mechanisms—exemplified by the DiGA model and US Medicare coverage—have proven critical by linking clinical efficacy (e.g., HbA1c reduction) and cost-effectiveness (e.g., CER thresholds) to reimbursement eligibility, creating sustainable incentives for adoption. Second, the integration of telemedicine and AI as a public health delivery model has demonstrated clear economies of scale: Empirical evidence from Brazil and Sweden confirms that this integration reduces healthcare costs at both patient and system levels while improving access and reducing complications. Third, policy focus on core system needs—including digital health innovation, data interoperability, privacy/security, and rural healthcare accessibility—has laid the groundwork for scaling by addressing technical and structural barriers to implementation.

Despite these advances, key challenges remain unresolved—with many being amplified by the process of scaling itself. Foremost is the global equity gap, with AI-enabled DHIs concentrated in HICs and largely underutilized in LMICs. Barriers in LMICs include limited reimbursement frameworks (only 5% incorporate cost-effectiveness), inadequate infrastructure (e.g., poor internet access and unaffordable devices), low digital literacy, and a lack of context-adapted tools—exacerbating disparities in T2DM management outcomes. More critically, scaling magnifies foundational issues that may be manageable in small-scale pilots but become systemic at scale: Privacy and security risks intensify as large volumes of sensitive health data are shared across interoperable systems, raising concerns about unauthorized access and data breaches; transparency deficits grow as complex AI algorithms (e.g., black-box ML models) are deployed widely, making it difficult for clinicians and patients to understand decision-making processes; algorithmic bias—rooted in unrepresentative training data (e.g., over-reliance on HIC populations)—is amplified, leading to inequitable care for marginalized groups (e.g., ethnic minorities and low-income patients) in scaled implementations; and computing power constraints emerge as a bottleneck in resource-limited settings, where LMICs often lack the infrastructure to support large-scale AI model training and real-time data processing.

Additionally, sustained implementation challenges are compounded by scaling: While edge AI and low-cost tools show promise for LMICs, policy design often fails to align with local evidence (e.g., India’s exclusion of cost-saving AI tools due to limited local data), and the logistical demands of rolling out interventions across diverse regions strain already constrained health systems. Finally, long-term alignment of policy, technology, and socioeconomic needs remains lacking—scaling requires not just technical solutions but also culturally tailored tools, community-based support, and public–private partnerships (PPPs) to address structural determinants (e.g., poverty and low education) that shape tool uptake and effectiveness; yet these elements are often afterthoughts in rapid scaling efforts.

In summary, scaling AI-enabled T2DM management is feasible when policies prioritize cost-effectiveness, integrate telemedicine–AI models, and address system-level enablers. However, the very act of scaling amplifies critical issues—privacy vulnerabilities, transparency gaps, algorithmic bias, computing power limitations, and implementation inequities—that threaten the sustainability and equity of these interventions. Resolving these amplified challenges requires a deeper examination of the ethical, technical, and systemic barriers that extend beyond policy and reimbursement frameworks.

## 5 Cross-cutting ethical dilemmas and governance risks

Across both clinical and population-level applications, AI deployment in T2DM management reveals recurring ethical and governance constraints, with the challenges of transparency and interpretability in AI-based clinical decision-making for T2DM becoming increasingly prominent. These challenges mainly stem from the “black box” nature of AI models, which limits doctors’ understanding of how algorithms arrive at specific diagnoses or treatment recommendations, thereby affecting clinical trust and widespread application (121).

### 5.1 Ethical dilemmas

#### 5.1.1 Transparency, interpretability, and clinical trust in AI-assisted T2DM decisions

##### 5.1.1.1 Why interpretability matters across CGM prediction, insulin dosing, and complication risk prediction

In clinical decision-making for T2DM, the transparency and interpretability of AI systems play a crucial role in specific clinical scenarios such as CGM prediction, insulin dosage recommendations, and complication risk prediction. Transparency emphasizes the clarity and understandability of the internal operating principles, algorithm logic, and data sources of AI models, while interpretability requires the system to provide clear and understandable reasons for its decisions, enabling clinicians to comprehend why and how AI reaches specific conclusions (122).

In the scenario of CGM prediction and blood glucose fluctuation monitoring, AI combined with CGM data can be used to predict future blood glucose trends, helping patients and doctors adjust treatment plans in a timely manner (23). However, the transparency of the internal mechanisms of AI models is crucial when predicting blood glucose fluctuations. Doctors need to understand which factors (such as carbohydrate intake during meals, physical activity, insulin injection time, and stress levels) are considered by the AI model when predicting blood glucose changes and their respective weights. For example, if an AI model predicts that a T2DM patient will experience a hypoglycemic event within the next 2 h, doctors will find it difficult to judge its reliability if the model lacks transparency and interpretability. However, if the model can explain that its prediction is based on the patient’s recent carbohydrate intake, the last insulin injection dose, and the hypoglycemic patterns observed in similar situations over the past few days, doctors can more confidently adopt this recommendation and guide the patient to take preventive measures.

In the scenario of personalized insulin dosage recommendations, AI systems can recommend the optimal insulin dosage based on patients’ blood glucose patterns, dietary habits, physical activity, and drug sensitivity data (123). However, the accuracy and safety of such recommendations also highly depend on the interpretability of the AI model. Doctors must understand the reasons for the AI’s recommendation of a specific dosage, such as whether it is based on the patient’s high fasting blood glucose level, postprandial blood glucose spikes, or increased insulin resistance recently. If the AI system only provides a dosage recommendation without explanation, doctors will be unable to evaluate its rationality, let alone adjust based on the patient’s individual circumstances (such as emotional fluctuations, stress responses, and other factors not captured by the model).

In the scenario of complication risk prediction, T2DM patients face risks of diabetic nephropathy, retinopathy, and cardiovascular disease—risks that AI predicts using clinical data, such as urine albumin-to-creatinine ratio (uACR) levels, HbA1c, and blood pressure (124). Both doctors and patients need to understand the basis for these risk predictions. For example, when an AI model predicts that a patient has a high risk of developing diabetic nephropathy, doctors need to know which specific biomarkers, genetic factors, or lifestyle habits have led to this high-risk assessment (122). Worryingly, adherence to uACR testing among T2DM patients is poor, which may lead to delays in the diagnosis and treatment of chronic kidney disease and further affect the quality and completeness of the training data for AI models (125). In such clinical scenarios with limited data quality, interpretability becomes particularly important as it can help doctors identify biases or uncertainties in model predictions.

##### 5.1.1.2 XAI approaches and evaluation in T2DM settings

To address the transparency and interpretability issues of AI in T2DM management, the field of XAI has emerged. This field aims to make AI models (e.g., DL) more interpretable and enhance the trust of stakeholders, including healthcare providers and patients. Notably, the establishment of such trust relies on the targeted application of XAI across diverse clinical scenarios in T2DM management. Core components of T2DM management—ranging from CGM prediction and personalized insulin dosage recommendation to complication risk assessment—demand clarity in the decision-making logic of AI models.

For CGM prediction in specific clinical scenarios of T2DM, inherently interpretable models can be developed. The decision logic of some AI models (such as DT or linear regression models [LRM]) is relatively simple and clear, and they can be regarded as “white box” models. Although they may not be as powerful as DL models in processing complex data, if their performance meets the standards in specific T2DM management tasks, these models can provide direct explanations (126). For example, a CGM prediction model based on a decision tree can directly show how different inputs of blood glucose, insulin, and diet lead to specific blood glucose trend predictions, allowing doctors to adopt the recommendation and guide the patient to take preventive measures. Such transparent explanations help enhance doctors’ trust in AI predictions in high-risk decision-making scenarios, where incorrect predictions may lead to serious clinical consequences.

For the transparency and interpretability issues in personalized insulin dosage recommendation, counterfactual explanations can be used. By slightly changing the input data to observe changes in model predictions, a deeper understanding of model behavior can be obtained (126). For example, a counterfactual explanation can state, “If the patient reduces carbohydrate intake by 10 grams or exercises for 20 minutes, the insulin dosage recommended by AI will decrease by 2 units.” At the same time, if an AI recommends increasing the dose of long-acting insulin before bedtime for a T2DM patient, the model can explain that this is because “the patient’s fasting blood glucose has been persistently high recently,” “the nighttime blood glucose monitoring data shows the presence of the dawn phenomenon,” and “the AI model believes that increasing this dose can effectively control morning hyperglycemia based on the patient’s insulin sensitivity assessment.” In this way, doctors can understand and discuss the adjustment plan with the patient. For this purpose, methods such as Shapley Additive Explanations (SHAP) or Local Interpretable Model-agnostic Explanations (LIME) can further identify that “high-carbohydrate diet” and “recent weight gain” are the main factors leading to the AI’s recommendation to increase the insulin dose (126), thereby facilitating doctors’ communication with patients and the formulation of more comprehensive intervention measures.

For the transparency and interpretability issues in complication risk prediction, feature attribution methods such as SHAP and LIME can also be used. By quantifying the contribution of each input feature to the model prediction, clinicians can identify which patient data have the greatest impact on the AI’s diagnosis or treatment recommendations. For example, in complication risk prediction, SHAP values can reveal the importance of HbA1c levels, blood pressure, or renal function indicators in predicting the risk of diabetic nephropathy (124). At the same time, if an AI model predicts that a T2DM patient has a high risk of developing diabetic nephropathy, interpretability technology can explain that this prediction is based on “the patient’s high microalbuminuria level,” “long-term poor blood glucose control (high HbA1c),” and “uncontrolled hypertension” (124). This not only helps doctors formulate targeted intervention measures (such as strengthening blood glucose and blood pressure management) but also improves patients’ awareness of risks and treatment adherence.

Future research should focus on developing XAI models oriented to clinical needs, integrating multimodal data and conducting large-scale clinical validation studies to address transparency and interpretability challenges. In conclusion, by integrating XAI technology and promoting interdisciplinary cooperation, AI is expected to play a safer, more effective, and more trusted role in clinical decision-making for T2DM in the future.

#### 5.1.2 Algorithmic bias and fairness mechanisms

Inherent biases in AI algorithms have become a key issue that may exacerbate existing healthcare inequalities, especially in terms of treatment outcomes for T2DM patients, which are particularly evident among different populations. Biases in AI algorithms may lead to misdiagnoses, inappropriate treatment recommendations, and ultimately affect the health outcomes of specific patient groups.

##### 5.1.2.1 Where bias originates

###### 5.1.2.1.1 Data collection and biased datasets

The most pervasive source of bias lies in training datasets that lack diversity, overrepresenting privileged groups while undercapturing the unique biological, environmental, and socioeconomic determinants of T2DM in marginalized populations.

First, the racial bias: Datasets dominated by European or North American cohorts fail to account for genetic heterogeneity and population-specific risk factors. For instance, a DL model trained on European T2DM patients (*n* = 5,000, 85% white) exhibited significant bias in UK-resident South Asian immigrants (*n* = 826): It underestimated the risk of diabetic nephropathy by 32% (AUC = 0.61 vs. 0.83 in white patients). This discrepancy arose from the model’s failure to integrate South Asian-specific traits, including higher baseline insulin resistance and dietary patterns (e.g., high refined carbohydrate intake) (33). The bias directly delayed albuminuria screening in 27% of South Asian patients, elevating their risk of end-stage renal disease by 18% (29).

Second, the age bias: Training data often underrepresent older adults, neglecting age-related physiological changes critical to T2DM management. An AI glucose management system deployed in 10 US clinics demonstrated this limitation: In patients ≥75 years (*n* = 342), its insulin dosage recommendations were 15% higher than clinically appropriate, leading to a 2.3-fold higher rate of severe hypoglycemia (4.2% vs. 1.8% in patients <65 years) (127). The root cause was training data in which only 12% of samples were ≥75 years, failing to capture age-related renal function decline and polypharmacy interactions that alter insulin metabolism (35).

Third, the regional and resource bias: Models developed in high-resource urban settings rarely incorporate rural-specific environmental or genetic factors. A G × E interaction model trained on urban Chinese cohorts (*n* = 10,232) performed poorly in rural populations (*n* = 2,150): It overestimated T2DM risk by 29% in rural patients with BMI < 25 kg/m^2^. This error originated from the exclusion of rural-specific variables, including heavy physical labor (which modulates metabolic demand), limited access to processed foods, and the ABCA1 69C > T polymorphism—more prevalent in rural Han Chinese and associated with T2DM susceptibility (24). Consequently, 31% of rural low-risk individuals were subjected to unnecessary lifestyle interventions.

Globally, over 70% of T2DM AI models are trained on European or North American datasets (24), yet LMICs—which account for 80% of global T2DM cases—are severely underrepresented in this training data; this imbalance embeds the priorities and phenotypes of privileged groups into algorithms, sidelining the needs of underserved populations and further amplifying health disparities. Such inherent biases in AI algorithms, rooted in both data and technical factors, exacerbate healthcare inequalities: Datasets dominated by European or North American cohorts fail to capture the G × E heterogeneity in LMICs (a gap amplified by limited local data collection capacity, a foundational technical challenge discussed in section 5.1), leading to biased risk predictions—for instance, a 32% underestimation of diabetic nephropathy risk in South Asian immigrants. Mitigating these issues requires constructing diverse datasets (to address data scarcity in LMICs, as noted in section 5.1) and developing fairness-aware algorithms.

###### 5.1.2.1.2 Algorithmic design and development

Even with balanced datasets, algorithmic design choices can inadvertently embed bias by prioritizing features more accessible to privileged groups (30). For example, AI-driven dietary intervention tools trained on Western diets may recommend low-carbohydrate plans incompatible with traditional Asian or African diets, reducing effectiveness for ethnic minority patients (68). Similarly, models optimizing for standard clinical metrics (e.g., HbA1c alone) may overlook context-specific markers—such as frequent postprandial glucose spikes in populations with high-carbohydrate diets—that better predict T2DM progression in marginalized groups.

Implicit biases of algorithm engineers further exacerbate this issue. For instance, downplaying the impact of structural barriers (e.g., food insecurity and limited healthcare access) in LMICs shifts blame for poor T2DM outcomes to individual behavior, rather than systemic constraints (30). This design choice undermines the utility of AI tools in resource-limited settings, where environmental factors often drive disease more strongly than individual choices.

###### 5.1.2.1.3 Model training and evaluation

The failure to incorporate fairness metrics (e.g., demographic parity and equalized odds) during validation allows models to prioritize overall accuracy at the expense of marginalized groups (128). A common concern in the field of medical AI is that AI systems may reflect and amplify human biases, thereby reducing the quality of their performance in historically underserved populations (129). For example, a T2DM risk prediction model trained on U. S. data underestimated risk in Hispanic populations by 30% due to unaccounted cultural dietary patterns and limited access to preventive care (33), directly linking algorithmic design to healthcare inequalities.

###### 5.1.2.1.4 Deployment and application context

Even rigorously tested AI models may exacerbate disparities in marginalized settings due to inadequate infrastructure or cultural mismatches. For example, AI systems requiring stable internet access or smartphones are inaccessible to 40% of rural populations in sub-Saharan Africa (33), while models lacking multi-language support or cultural adaptation fail to serve non-English-speaking minority groups (115). Reimbursement policies that prioritize AI-driven tools further widen inequalities—patients without insurance coverage for DHIs are denied access to personalized AI recommendations, reinforcing the “digital health divide” (117).

##### 5.1.2.2 Clinical and population impact pathways

**Figure 5:**
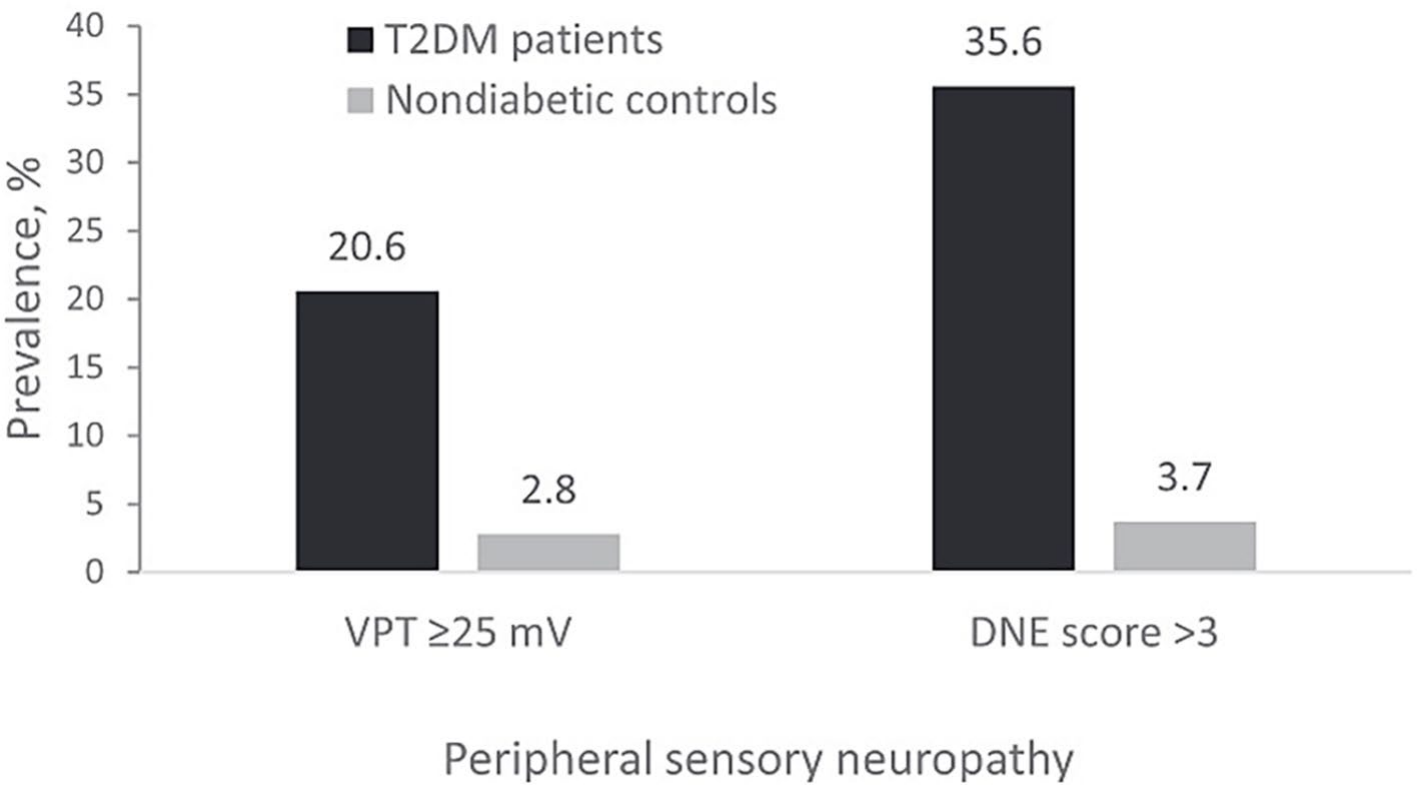
Prevalence of peripheral sensory neuropathy among study participants. Figure adapted with permission from Agyekum and Yeboah (131).

**Figure 6:**
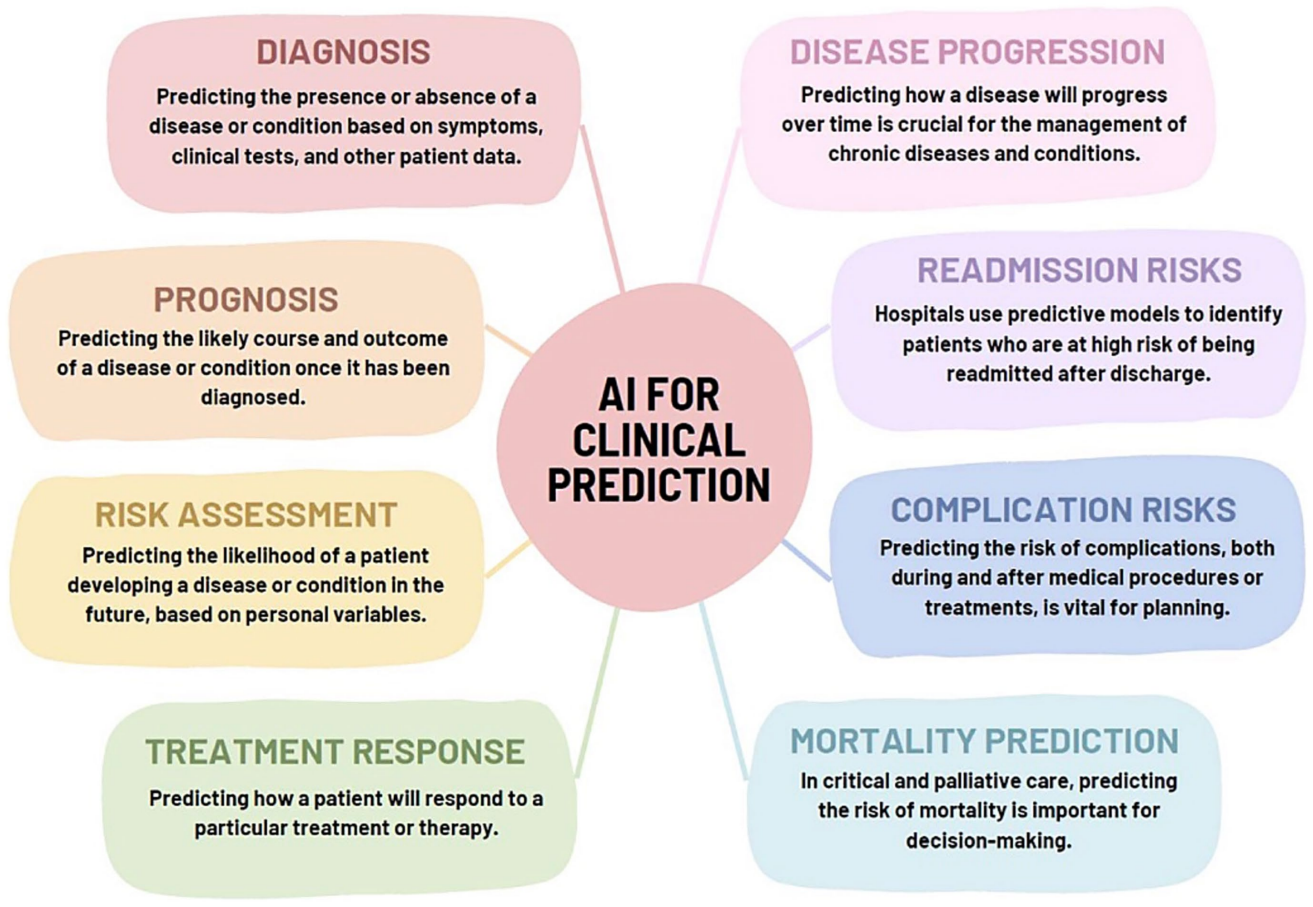
AI supports eight domains of clinical prediction. Figure adapted with permission from Khalifa and Albadawy (132).

AI bias may affect the treatment outcomes of T2DM patients in the following aspects:

(1) Diagnostic accuracy: AI-assisted diagnostic tools may have different accuracies among different demographic groups. For example, if a model does not fully learn T2DM-specific biomarkers [such as HbA1c levels (130)] in different racial or age groups, it may lead to missed diagnoses or misdiagnoses in certain groups, thereby delaying treatment. A study by scholars such as Jennifer A. Agyekum shows that peripheral sensory neuropathy is a common complication of T2DM. If AI fails to effectively identify subtle neurophysiological differences in patients of different ethnic groups, it may affect the accuracy of its early diagnosis. As shown in Figure 5, peripheral sensory neuropathy is a common complication of T2DM, with its prevalence in T2DM patients significantly higher than that in non-diabetic control groups—this holds true whether measured by vibration perception threshold (VPT ≥ 25 mV) or diabetes neuropathy examination (DNE score >3) (131). AI models must consider the physiological differences of different populations to avoid potential biases.
(2) Treatment plan recommendation: Population-specific clinical characteristics and treatment responses further amplify the impact of AI bias. Gesine van Mark et al. highlighted that older adult T2DM patients exhibit distinct clinical manifestations (e.g., age-related renal function decline and polypharmacy) and treatment needs compared to younger patients; AI-driven treatment recommendation systems that lack optimization for age-related heterogeneity may propose suboptimal strategies (e.g., overestimating insulin dosages) based on biased training data (127). Similarly, AI-powered dietary interventions—often trained on Western dietary patterns—may conflict with traditional dietary habits of Asian, African, or Latin American populations when analyzing CGM data, reducing intervention effectiveness (68). As shown in Figure 6, Khalifa Mohamed et al. elaborate on the eight core domains of clinical prediction supported by AI, among which diagnosis, prognosis, and treatment response are closely related to T2DM management. They further noted that AI bias in these domains disproportionately harms underrepresented groups, as models frequently fail to capture the complexity of their disease trajectories (132). A striking example of therapeutic bias is observed in a US multi-center study (*n* = 12,000 T2DM patients): AI-driven medication recommendations showed significant racial disparity—Black patients with identical clinical profiles (HbA1c = 8.0%, BMI = 30 kg/m^2^) were 2.1 times less likely to receive GLP-1 RA than white patients (129). Over 5 years, this discrepancy was associated with a 27% higher myocardial infarction rate in Black patients, directly linking algorithmic bias to adverse cardiovascular outcomes (26).
(3) Exacerbation of health inequalities: AI bias may exacerbate existing healthcare gaps, leading to underserved groups (such as ethnic minorities, low-income populations, and people in remote areas) receiving poor treatment outcomes and poor performance of AI models in these groups (133). For example, in the field of cardiovascular health, AI has been considered a tool to detect and reduce racial and ethnic inequalities, but if AI itself has biases, it may instead exacerbate these inequalities (30). At the same time, although telemedicine is regarded as a potential strategy in T2DM management, its effectiveness still needs further research. If the promotion of AI exacerbates the digital divide, it may further widen healthcare inequalities (134).

##### 5.1.2.3 Mitigation strategies and reporting standards

To ensure the fairness and effectiveness of AI in T2DM management, a multi-dimensional strategy framework should be established, covering dataset construction, algorithm development, post-deployment monitoring, and personalized application, while integrating fairness considerations into the full lifecycle of AI systems.

**Figure 7:**
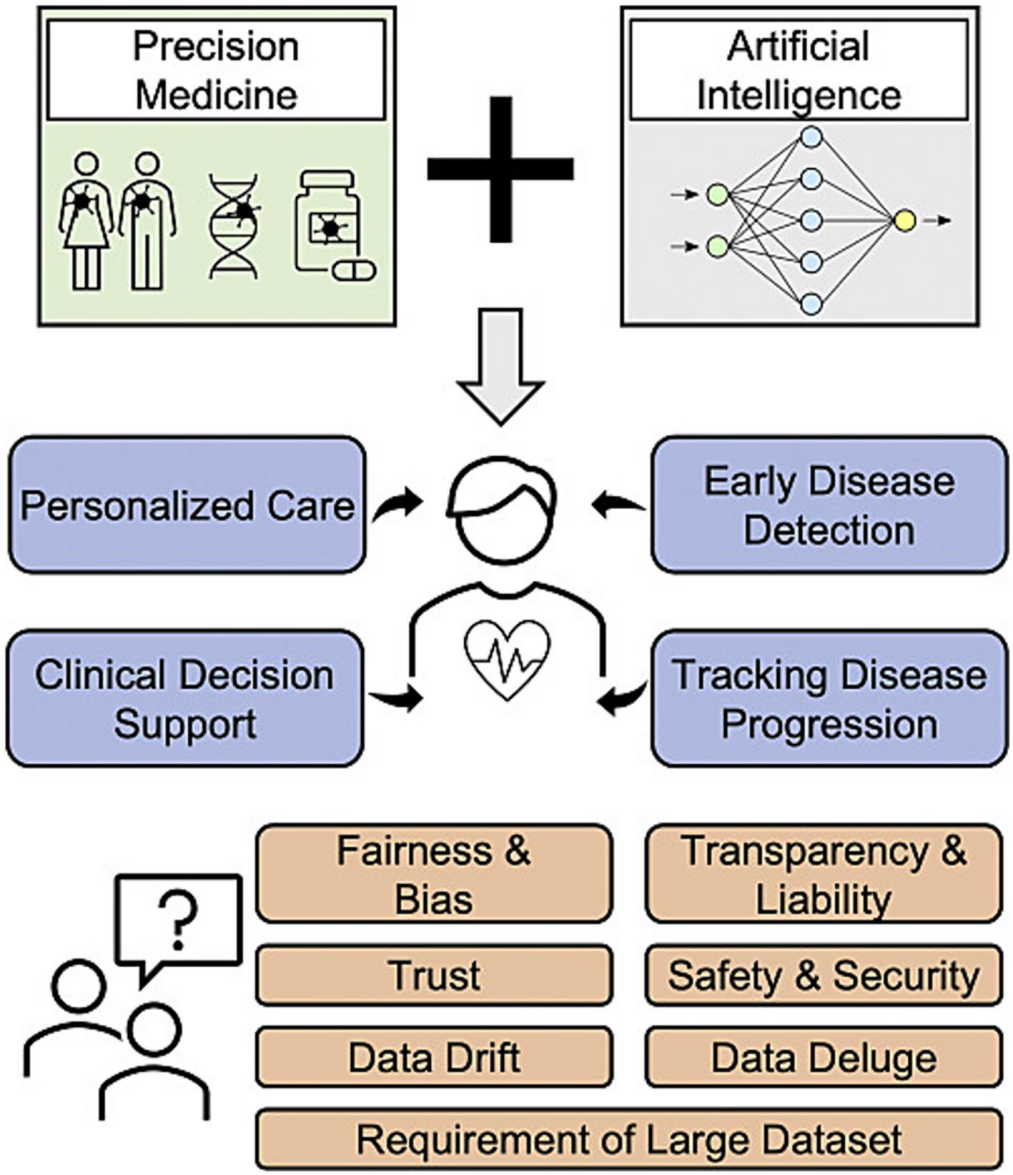
Overview of incorporation of artificial intelligence into precision medicine. Figure adapted with permission from Shandhi and Dunn (35).

First, use diverse and representative datasets, which are the basis for mitigating AI bias. Researchers and developers need to collect comprehensive and representative datasets from different races, ages, genders, and socioeconomic backgrounds, such as redesigning datasets to achieve inclusiveness and clearly defining training objectives (30). Second, develop algorithms that can proactively identify and mitigate biases and perceive fairness, using technologies including fairness constraint optimization and adversarial debiasing (129). At the same time, during the algorithm development stage, train algorithm writers to avoid exacerbating existing inequalities. Third, after the deployment of AI systems, continuously monitor and audit their performance across different populations, implement bias audits and dataset monitoring, and retrain as needed (30). Finally, AI applications should aim to achieve true personalized medicine: by integrating pharmacogenomics and pharmacoepigenomics to deeply understand individual patients’ drug response characteristics (135), combining multimodal data (e.g., EHRs) with AI technologies (e.g., RL), personalized multimorbidity management for T2DM can be realized to improve patient health outcomes (136)—notably, as shown in Figure 7, studies by scholars such as Md Mobashir Hasan Shandhi visually demonstrate the integration framework of precision medicine and AI in healthcare, highlighting their synergistic impact on T2DM personalized management while also pointing out key challenges such as algorithmic fairness and bias that need to be addressed (35). Meanwhile, fairness indicators should be incorporated as core considerations in the early design and development stages of AI and digital health tools; in AI performance evaluation, attention should not only be paid to overall accuracy but also to performance across different subgroups (e.g., populations with different ethnicities, socioeconomic statuses, and geographical locations), with priority given to improving the accuracy of underperforming subgroups (128). Additionally, the interpretability and transparency of AI decision-making processes should be enhanced to enable users and clinicians to understand the rationale behind AI recommendations, thereby building trust in AI systems (111).

In conclusion, the inherent bias problem of AI in T2DM management cannot be ignored. Future research and practice should focus on developing fair, transparent, and responsible AI systems to ensure that they can provide high-quality medical services to all populations and truly achieve fairness in healthcare.

### 5.2 Data privacy, security, and governance risks in AI-enabled T2DM

Data in the public health sector typically contains a wealth of sensitive personal health information, including diagnoses, treatments, medication records, and lifestyle habits. Its collection, management, and utilization drive revolutionary progress in enhancing medical quality and improving public health outcomes. However, the leakage or abuse of such sensitive information may lead to personal discrimination, economic losses, and even physical harm. Consequently, data and privacy protection serve as the cornerstone for building public trust and ensuring the effective operation of health information systems.

#### 5.2.1 Threat landscape in multi-source health data (re-identification, breach, key management, and secondary use risks)

**Figure 8:**
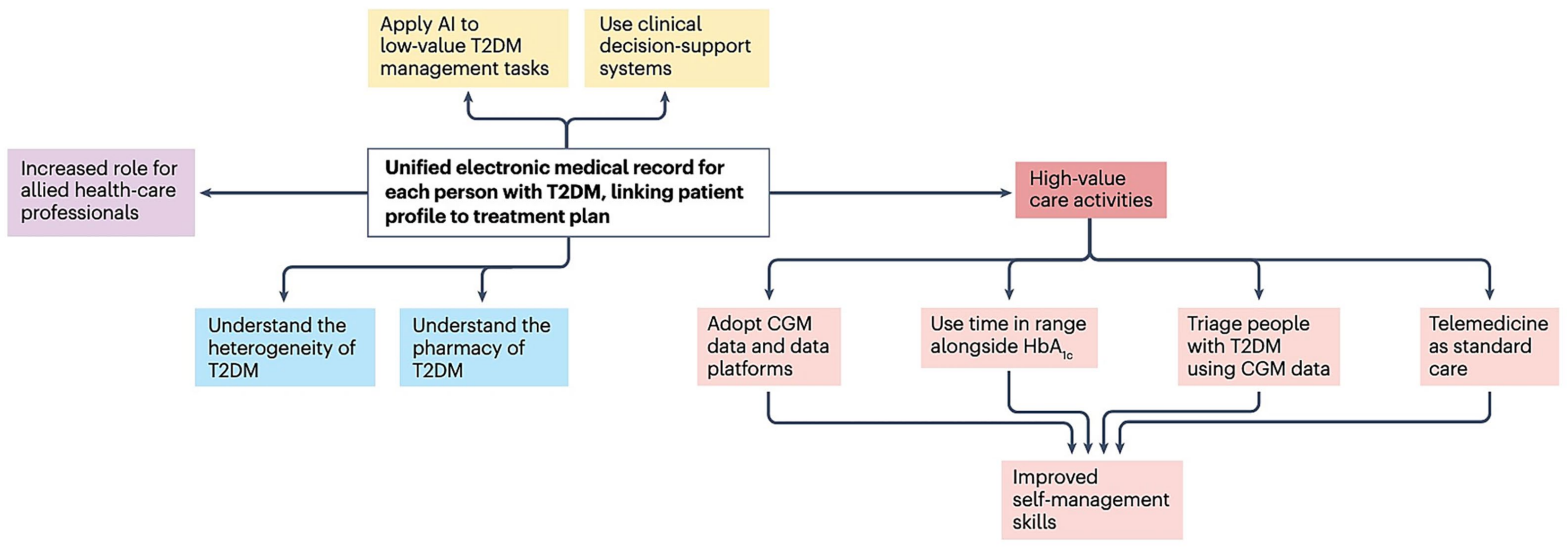
The unmet need for change in healthcare services for effective management of people with type 2 *diabetes mellitus*. Figure adapted with permission from Ajjan et al. (23).

**Figure 9:**
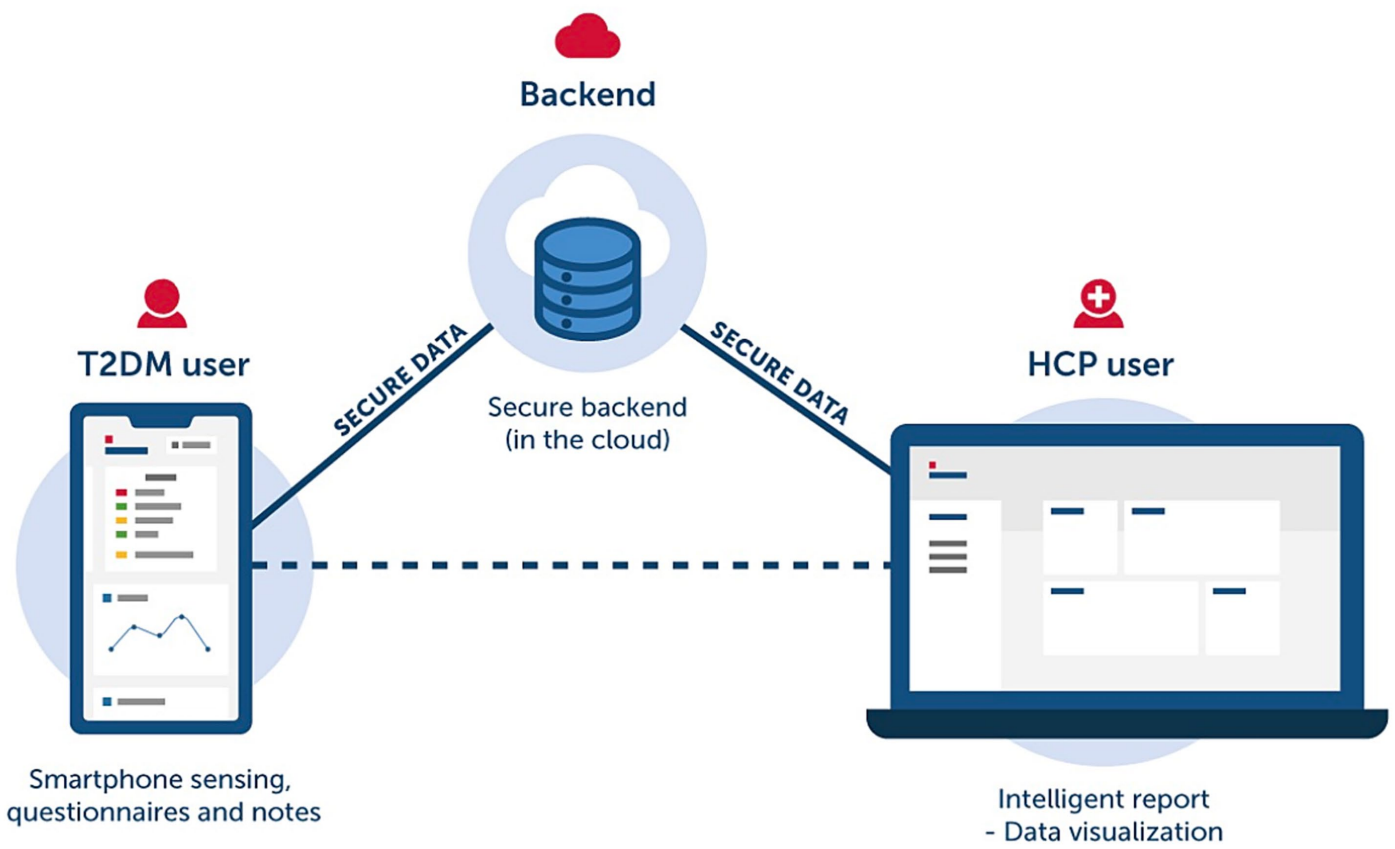
The overall architecture of the DiaFocus system comprising the CARP cloud-based backend, the DiaFocus smartphone app, and the clinical web application with associated reports. Figure adapted with permission from Bardram et al. (140).

With the widespread application of digital health tools, the privacy and security of T2DM patients’ multi-source health data face a diverse threat landscape, with key risks manifesting in the following dimensions:

(1) Data Sensitivity and Re-identification Risk: T2DM patients’ data encompasses high-sensitivity information, including personal identity information and granular health data (e.g., glucose monitoring records and genetic data). This sensitivity renders the data vulnerable to re-identification—even when anonymization measures are applied, the risk of linking de-identified data back to individuals persists, with leaks potentially causing severe harm to patients (137). Notably, the widespread adoption of CGM further amplifies this risk by generating massive volumes of such sensitive personal health data, making data and privacy protection imperative for effective T2DM management. As shown in Figure 8, scholars like Ramzi A. Ajjan visually present the unmet need for change in healthcare services for effective T2DM management, highlighting “adopting CGM data and data platforms” as a key component of optimized strategies. This underscores the critical need to safeguard sensitive CGM data amid its integration for precise care (23).
(2) Data Breach Risk: Multi-source data integration (e.g., merging EHRs, wearable device data, and mHealth platform data) introduces significant security vulnerabilities, creating attack surfaces for unauthorized access and data breaches (110). Real-world incidents highlight this risk: A 2024 breach of a global mHealth platform exposed 1.2 million T2DM patients’ genetic and glucose data, despite the implementation of end-to-end encryption, due to flawed key management and inadequate security auditing (138). As detailed in Table 3, such data breach risks stem from security vulnerabilities in multi-source data integration, and corresponding safeguards like blockchain technology can mitigate unauthorized access by leveraging its decentralized and tamper-proof characteristics. Additionally, traditional centralized data collection (a common practice for training ML models) concentrates data assets, further elevating the risk of large-scale breaches and associated compliance violations (105).
(3) Key Management Vulnerability: As a critical component of data encryption (e.g., for end-to-end protection of sensitive health data), inadequate key management emerges as a distinct attack vector. Deficiencies in key storage, distribution, or access control can undermine even robust encryption protocols, leading to unauthorized data exposure—evidenced by the 2024 mHealth breach (138).
(4) Secondary Use and Abuse Risk: Sensitive T2DM patient data (e.g., personal speech samples collected via digital health tools for adherence monitoring) is at risk of unauthorized secondary use or abuse. Without strict access controls and usage audits, such data may be accessed, shared, or exploited for non-clinical purposes without patient consent (139).
(5) User Trust Erosion as a Consequential Risk: Patients’ concerns about the aforementioned risks (e.g., breach and unauthorized secondary use) directly erode their trust in digital health tools, reducing their willingness to share data or engage with these platforms. This further compromises the utility of multi-source data while exacerbating existing security-related challenges (138). Relevant solutions and practices offer mitigation insights; for example, scholars like Jakob E. Bardram developed DiaFocus, a mobile health sensing application for long-term dynamic T2DM management. As shown in Figure 9, its overall architecture comprises the Copenhagen Research Platform (CARP) cloud-based backend, the DiaFocus smartphone app, and a clinical web application with associated reports, supporting an adaptive collaborative care model through “secure data transmission” to protect patient information confidentiality (140). Moreover, given that mHealth apps typically collect diverse data (weight, height, blood pressure, steps, resting heart rate, geographic location, physical activity) stored on developers’ servers or shared with family members, strict authentication mechanisms and user control over data collection/sharing are critical to mitigating breach and abuse risks (141).

**Table 3:** Risks and challenges in patient data privacy and security and corresponding strategies.

Risk/challenge	Control point	Evidence/standard
Large volume and high sensitivity of data	Differential privacy technology	Add noise to data to obscure individual records, retain the overall statistical characteristics of the dataset, and protect individual privacy.
Security vulnerabilities in data integration	AI-driven encryption technology	Dynamically adjust encryption strategies according to the role and authority of accessing users, effectively preventing security vulnerabilities in data integration.
Increased leakage risk due to centralized data	Federated learning technology	Allow AI models to be trained on local devices without uploading raw data to a central server, significantly reducing the risk of data leakage.
Easy unauthorized abuse of data	Blockchain technology	Utilize the decentralized, tamper-proof, and transparent characteristics of blockchain to prevent unauthorized access; de-identify data before data sharing and analysis to prevent AI models from generating bias due to data homogeneity.
Potential bias in AI models		
Patients’ concerns about digital health tools	Informed consent and patient empowerment	Ensure that patients fully understand how their data is used by digital health systems and have the right to give or withdraw consent.

#### 5.2.2 Technical safeguards

Addressing the above challenges requires adopting multiple technical strategies to ensure the privacy and security of T2DM patients’ data. A study by scholars such as Dr. Prachi Tripathi explored the dual problem of making full use of data to enable most AI models to function while ensuring patient privacy, proposing privacy protection technologies such as differential privacy. By adding noise to data to obscure individual records while retaining the overall statistical characteristics of the dataset, the privacy of individuals is protected (137). Additionally, anonymization and pseudonymization can be applied alongside differential privacy; through data desensitization technologies (e.g., deleting or modifying personal identifiers), personal information is made difficult to identify while retaining the value of data for research and analysis (142). For instance, medical data systems can assign random personal codes to replace real patient names for public retrieval to ensure patient privacy. However, it is important to note that there are differences between pseudonymized data and anonymized data, and not all anonymization technologies comply with General Data Protection Regulation (GDPR) standards (143).

AI-driven encryption technology proposed by scholars such as Dilli Ganesh V creates a dynamic encryption environment, adaptively encrypting data according to data sensitivity and user type. Data of different sensitivity levels can adopt different encryption strengths, and the system can dynamically adjust encryption strategies according to the role and authority of accessing users, effectively preventing data leakage and security vulnerabilities during data integration (144). As a basic method for protecting data privacy, encryption ensures the confidentiality of data during transmission and storage (142), and searchable encryption technology further enables searching without decrypting data, realizing data retrieval under privacy protection (145).

FL proposed by scholars such as Ayyappan G allows AI models to be trained on local devices without uploading raw data to a central server. This decentralized approach significantly enhances patient privacy protection, reduces the risk of data leakage, and ensures compliance (105). FL was prospectively validated in a multi-center study (five European hospitals) for T2DM risk prediction, achieving an AUC of 0.76 without centralizing sensitive data. There was no increase in data breaches, and the model identified high-risk patients who benefited from early insulin initiation (HbA1c reduction of 0.7%). It was scaled in the EU via the “AI Act” regulatory framework but failed in LMICs due to high bandwidth requirements (80% of rural health centers cannot support FL) (105). FL reduces model accuracy by 15–20% vs. centralized training, compromising clinical utility in low-data settings (137). To further reinforce data security in FL scenarios, access control mechanisms can be integrated; through models such as Attribute-Based Access Control (ABAC) and Role-Based Access Control (RBAC), only authorized personnel are allowed to access sensitive data involved in local model training or parameter sharing (142).

Scholars such as Mohamed Maher proposed blockchain technology, which utilizes the decentralized, tamper-proof, and transparent characteristics of blockchain to ensure data integrity, prevent unauthorized access, and provide audit trails. In this regard, relevant studies propose building a blockchain-based digital health data market to enhance patients’ participation in their health data repositories. Meanwhile, before data sharing and analysis, data should be de-identified by removing or encrypting personal identity information to prevent AI models from generating bias due to data homogeneity (138). A blockchain-based EHR system was validated in 300 T2DM patients in Dubai, reducing data leakage incidents to zero over 2 years while maintaining data integrity. Improved care coordination reduced diabetes-related complications (e.g., retinopathy screening compliance increased by 40%). However, it was limited to high-resource settings (e.g., the UAE and Singapore) due to infrastructure costs and failed in rural India due to a lack of technical support (60% of facilities abandoned the system within 6 months). A blockchain pilot for CGM data sharing in Kenya was discontinued due to low user trust (patients feared data misuse) and high maintenance costs (138). Additionally, encryption technology (e.g., end-to-end encryption) can be combined with blockchain to further secure data transmission and storage within the blockchain ecosystem, and access control can be implemented to restrict permissions for data viewing and modification on the blockchain (142, 145).

**Figure 10:**
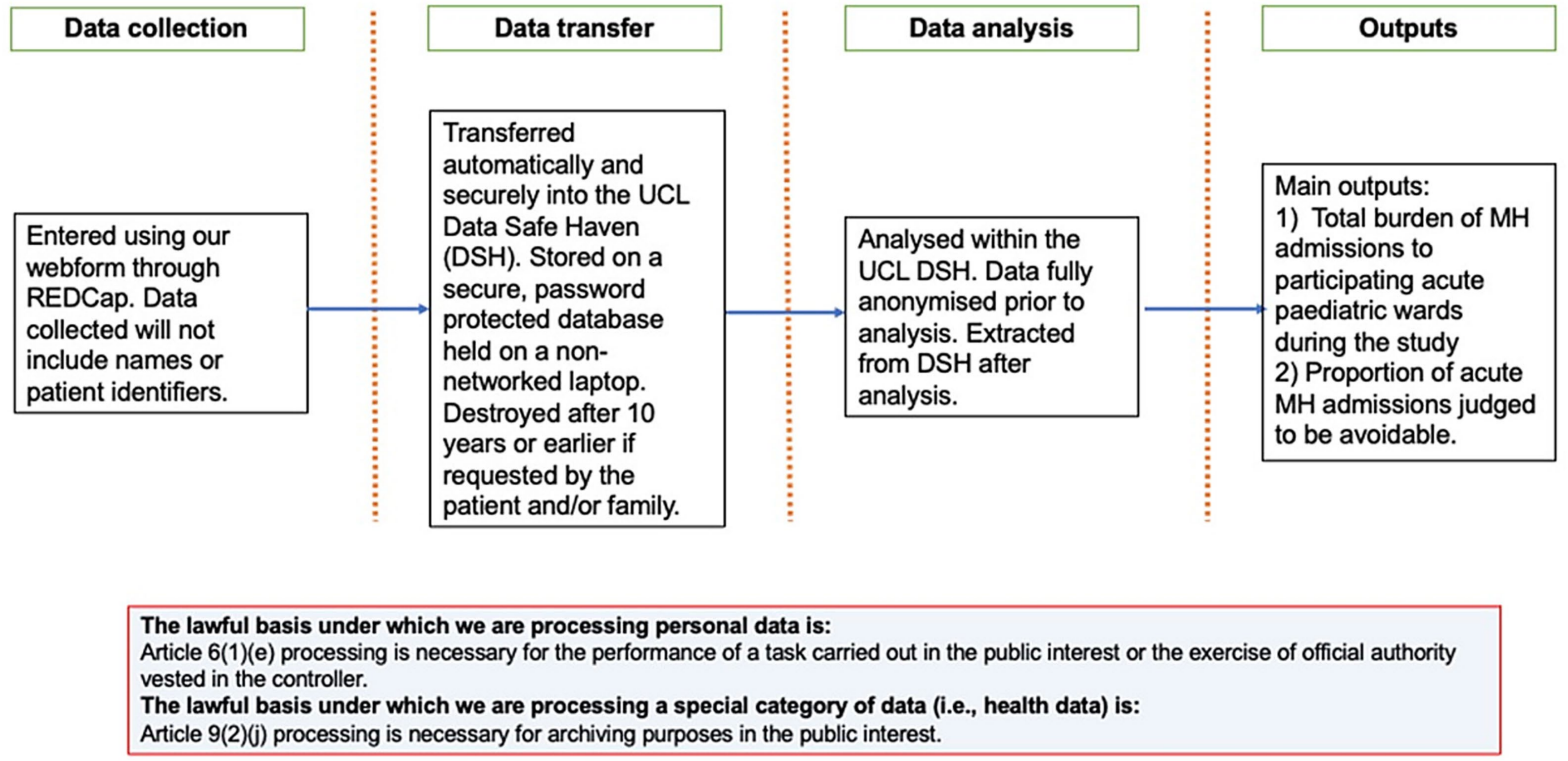
WP2 data flow diagram. MH, mental health; UCL, University College London; WP, work package. Figure adapted with permission from Hudson et al. (147).

Additionally, scholars such as Kedar Mohile propose that informed consent and patient empowerment—ensuring patients fully understand how AI-driven digital health systems use their data and retain the right to grant or withdraw consent—should be prioritized; this can be achieved via clear communication and user-friendly interfaces to enhance patients’ autonomy and control over their health data (146). Equally crucial is enhancing public awareness and participation: Data and privacy protection goals are advanced by raising public awareness of the importance of personal data privacy, encouraging active engagement in protection efforts, and upholding patients’ core data rights (including informed consent, access, correction, and deletion). As shown in Figure 10, scholars such as Lee Duncan Hudson illustrate this through a detailed medical research data processing framework (WP2 data flow diagram): Patient names/identifiers are collected but destroyed before data transmission, after which data is securely transmitted to a protected database, anonymized, retained for ≤10 years or deleted per patient request, and analyzed in a secure environment using fully anonymized data. This framework aligns with legal bases for public interests and processing special-category T2DM health data for medical purposes (147).

#### 5.2.3 Limits and trade-offs

With the widespread application of digital health tools, the privacy and security protection of T2DM patients’ data face prominent trade-offs between technical feasibility, implementation cost, and clinical utility. In terms of privacy-preserving technology trade-offs: (1) FL: As a decentralized data processing paradigm, FL avoids direct data aggregation but imposes strict infrastructure requirements. It demands communication bandwidth up to 10 times higher than centralized model training, coupled with compatible hardware support. This cost barrier makes FL unaffordable for 80% of rural health centers in LMICs, severely limiting its scalability in resource-constrained T2DM care scenarios (105). (2) Differential Privacy: While differential privacy achieves individual identity protection by injecting noise into data, it creates an unavoidable accuracy trade-off. When the noise intensity reaches the threshold required to prevent re-identification, the accuracy of T2DM-related health prediction models decreases by 15–20%, directly compromising the models’ clinical applicability (e.g., glucose trend prediction and complication risk assessment) (137).

In terms of real-world validation of trade-off gaps, real-world data security incidents further highlight the practical limitations of current protection measures. As mentioned earlier, a global mHealth platform experienced a data leak, exposing genetic information and glucose monitoring records of 1.2 million T2DM patients in 2024. Notably, the platform had already deployed end-to-end encryption—a common privacy-preserving measure—yet the incident still occurred, reflecting the mismatch between theoretical protection effects and actual implementation capabilities, and further verifying the difficulty of balancing protection intensity and practical security (138). Additionally, patients’ lingering concerns about data security and privacy (a direct consequence of unresolved trade-offs) reduce their willingness to share real-time T2DM data (e.g., wearable device records), creating a secondary conflict between data availability for model optimization and user trust (138).

In conclusion, data protection in the public health field still faces many challenges (e.g., data formats generated by different medical institutions and devices are inconsistent, leading to difficulties in data integration and sharing and increasing the complexity of privacy protection). The risks of emerging technologies cannot be ignored either. AI is increasingly used in the medical field, but AI models may have privacy leakage risks, such as inferring sensitive information through models (104). In the future, data protection in the public health field will develop in a more intelligent and refined direction. The development of AI-assisted medicine and the advent of the IoT and 5G/6G communication era will bring a more humanized medical environment and personalized services (104). This requires continuous research and development of innovative privacy protection technologies, while strengthening international cooperation, learning from the experiences and best practices of various countries in data protection, and building a more comprehensive global health data governance system.

## 6 Governance, regulation, and policy frameworks

### 6.1 Governance challenges in regulating AI-enabled digital health for T2DM

Addressing these systemic constraints ultimately requires policy-level interventions that align innovation with regulatory oversight and health system capacity. However, current public health policies still face numerous limitations and obstacles in advancing the application of AI and digital health tools, meaning policymakers must focus on core constraints including data governance and privacy protection, lagging regulatory frameworks, insufficient infrastructure and resources, and ethical considerations.

#### 6.1.1 Data governance and privacy compliance

The sensitivity of health data brings strict data protection requirements. In low-resource government health systems, effective health data governance is crucial for AI innovation. The application of AI and IoT in medical information management also requires sound data collection, data quality, data accuracy, and data governance strategies. The incomplete legal framework of current policies regarding health data access, exchange, and security constitutes a major obstacle. For example, although digital tools can significantly improve medical quality and promote access to medical services for vulnerable groups, if the implementation and management of digital healthcare are improper, it may inadvertently exacerbate health disparities (148). This requires that data protection and privacy issues must be fully considered in data sharing and AI applications, especially when processing personal health information.

#### 6.1.2 Regulatory lag and adaptive approval pathways

The rapid iteration of AI technology creates a mismatch between existing policies and its update speed, while the approval and application of digital health tools demand clear, flexible regulatory pathways to balance innovation and risk control. The lack of dedicated AI regulatory frameworks introduces uncertainty, ultimately hindering the widespread adoption of digital health tools: This challenge is evident across diverse contexts: For instance, while Canada has implemented extensive AI governance measures, it still faces difficulties in effectively managing the potential impacts of AI systems (149). Nigeria similarly confronts a mix of opportunities and obstacles when integrating AI into health communication policies (150). Notably, Canada has taken targeted steps to address such gaps through its AI and Data Act (2023), which mandates AI tool developers to submit “fairness impact assessments.” A practical application of this requirement is the adjustment of the Canadian Diabetes Association’s risk prediction model—. By incorporating Indigenous-specific risk factors (e.g., residential school history-related stress markers), the model reduced missed diagnoses in First Nations populations by 33% (149), illustrating how adaptive regulatory measures can mitigate uncertainties while advancing equitable AI deployment in diabetes care.

#### 6.1.3 Resource constraints as governance issues

The effective implementation of AI and digital health tools in the public health field requires strong digital infrastructure support. However, as discussed in section 5.1, foundational challenges—including human capacity gaps—hinder AI deployment in LMICs. Beyond the digital infrastructure-related gaps outlined in the foregoing, import tariffs on digital health tools (up to 25% in Nigeria and 30% in India) increase device prices by 30–50% (103), making them unaffordable for most healthcare facilities. Governments in LMICs lack funds to subsidize these costs—only 5% of national health budgets in sub-Saharan Africa are allocated to digital health (11). Furthermore, the lack of local manufacturing capacity (a challenge distinct from the infrastructure constraints addressed in section 5.1) means that replacement parts and technical support are unavailable, leading to high device failure rates: 60% of donated wearables in rural Kenya become inoperable within 6 months (114). For example, in South Asia, although health data science helps alleviate the burden of cardiovascular disease management, digital health technologies still face challenges related to medical service accessibility and affordability (separate from the infrastructure barriers detailed in section 5.1) (151). In addition, professional talent is also scarce. LMICs have only 0.1 digital public health specialists per 100,000 people (vs. 5 in HICs), leaving healthcare workers unable to operate or maintain AI tools (152). To address this, initiatives like the World Health Organization’s (WHO) “Digital Health Academy” train CHWs in basic AI tool use, and partnerships between LMIC universities and HIC tech companies (e.g., Google’s AI for Global Health program) are cultivating local talent (29).

#### 6.1.4 Ethical governance and social license

The application of AI in healthcare and public health raises multiple ethical issues, including algorithmic bias, fairness, transparency, and responsibility attribution. For example, AI systems may structure social, political, and economic determinants of health into invisible algorithms (153). At the same time, public trust in AI and its impact on social fairness are directly related to the social acceptance of digital health tools. If relevant policies fail to fully address these ethical concerns, it may lead to public resistance and affect the popularization of AI tools.

#### 6.1.5 Cross-sector coordination and implementation governance

The formulation of public health policies often involves multiple sectors and stakeholders, and the effective integration of AI and digital health tools requires close cooperation and policy coordination between the government, medical institutions, technology companies, and academia. However, current policies lack effective coordination mechanisms, leading to fragmentation and inefficiency (154). In addition, the Spatial Data Infrastructure (SDI) framework plays a key role in integrating and sharing geospatial data, but its governance and policies in regions such as Africa and Asia still face challenges (108).

### 6.2 Policy and regulatory recommendations: a governance framework for safe and equitable scale-up

To ensure the legal, compliant, and safe application of the integration of AI technology and public health policies, it is crucial to establish a sound regulatory framework. This framework not only needs to address the challenges brought by the rapid development of technology but also balance innovation and risk, especially in the application of digital health tools.

#### 6.2.1 Privacy-by-design and accountable data stewardship

A sound data governance framework, underpinned by a robust legal and regulatory system, is the basis for the integration of AI and public health policies. Such a system should clarify rules for the collection, use, storage, and sharing of health data, and stipulate responsibilities and penalties for data leakage: For instance, China’s *Personal Information Protection Law* serves as an important milestone in addressing health data protection challenges amid digital transformation (155), while internationally, the GDPR provides a strong data protection framework—though its application to health research still requires balancing public interests and personal control (156). Additionally, public health data governance must emphasize the “principle of minimum necessity,” meaning only the minimum volume of data required to achieve a specific purpose is collected and used, with re-identification risks in records kept as low as possible (157)—thus striking a balance between the risks of privacy leakage and the potential value of secondary data use.

Beyond legal foundations, effective data governance encompasses four key pillars. First, formulate clear data sharing and use specifications. Public health institutions should clarify data sharing agreements with data providers (such as hospitals and research institutions), specifying detailed processes and standards for data collection, storage, processing, use, and destruction, including specific requirements for data anonymization or pseudonymization to protect personal privacy (152). Second, implement the Privacy by Design principle. Embed privacy protection mechanisms in the early design stage of AI systems and digital health tools to ensure that data protection runs through the entire life cycle (152). This means that privacy protection measures should be the default setting of the system rather than an additional option, from data collection to algorithm development and application deployment. Third, establish a transparent data use audit mechanism. Allow audits of how AI systems use health data to ensure that data use complies with established policies and legal regulations (158). Finally, strengthen cross-sectoral data interoperability. Encourage the establishment of unified data standards and interfaces to promote data interconnection between different public health systems and medical institutions (while ensuring the security and compliance of data transmission) (108); regulatory authorities should also explore cross-border interoperability frameworks to enable seamless data flow across geographical regions while upholding data privacy and security.

Notably, to address the lack of regulatory capacity in LMICs, simplified, culturally adapted data privacy frameworks should be developed—Examples include Rwanda’s 2022 Data Protection Law, which exempts anonymized T2DM cohort data from strict cross-border transfer restrictions while mandating local storage of raw genetic data (this enabled a multi-center study on rural T2DM in East Africa involving 10,000 patients without compromising privacy) (159), and India’s Digital Personal Data Protection Act 2023, which classifies T2DM health data as “sensitive personal data” and requires explicit consent for AI analysis (telemedicine platforms like “Practo” now provide audio consent options for low-literacy patients, reducing data breaches by 60% since implementation) (160). These policy cases align with the privacy protection strategies outlined in Table 3, emphasizing the combination of legal frameworks and technical safeguards to balance data utility and patient privacy.

#### 6.2.2 Adaptive regulation and lifecycle oversight

The rapid iteration of AI technology requires a regulatory framework with a high-flexibility and forward-a looking approach. Given the complexity and rapid development of clinical AI, traditional centralized regulatory models are difficult to adapt, In this regard, scholars such as Trishan Panch proposed a distributed regulatory approach, involving multiple stakeholders (including developers, medical institutions, patients, and regulatory authorities) in regulation to better adapt to the heterogeneity of local health systems and address issues such as data drift, while the framework itself should remain flexible to adjust with technological advancements and the emergence of new evidence (160).

First, establish a dynamic risk assessment and classification system. Formulate hierarchical regulatory requirements and approval processes based on the risk level of AI applications in public health (e.g., from low-risk health education tools to high-risk diagnostic assistance systems), with high-risk AI applications subject to stricter reviews, including clinical verification and post-marketing monitoring (153). Regulatory authorities should establish clear, operable market access standards, covering detailed requirements for the design, development, data management, and verification of medical AI products—exemplified by the U. S. FDA’s pre-market review process, which mandates manufacturers to provide sufficient evidence of safety and effectiveness (159), and China’s National Medical Products Administration (NMPA), which launched a “fast-track approval pathway” for AI diabetes tools in 2024. For instance, the “AiTang” CGM analytics platform (integrating explainable AI to show patients how diet affects glucose trends) was approved in 6 months after demonstrating a 0.5% HbA1c reduction and is expected to cover 500,000 patients nationwide by 2025 (161).

Second, implement the regulatory sandbox or innovation center model. Build a controlled environment to test and verify innovative AI digital health tools within a limited scope while collecting real-world data under the guidance of regulatory authorities to evaluate their effectiveness, safety, and potential risks—this accelerates technology translation without compromising safety (161). Notably, regulatory sandboxes can incorporate cost-effectiveness as a key evaluation indicator, such as the UK’s Medicines and Healthcare products Regulatory Agency (MHRA), which established a cost-effectiveness fast track for AI tools, requiring developers to submit 1-year real-world data on CER. An AI insulin dosage optimizer, for instance, was approved in 6 months after demonstrating a CER < €20,000 per QALY (161).

Third, implement post-marketing monitoring and continuous evaluation. Establish a sound post-marketing monitoring system for approved AI digital health tools, continuously collect performance data in actual applications, and conduct regular risk–benefit evaluations (153), including adverse event reporting, performance drift detection, and long-term monitoring of algorithm fairness.

Finally, clarify legal responsibilities and accountability mechanisms. For errors or damages caused by AI decisions, clearly define the responsibilities of developers, users, medical institutions, and regulatory authorities, and establish corresponding legal recourse and compensation mechanisms (159). This requires robust audit trails and incident reporting systems to facilitate investigation and learning from adverse events (106). For LMICs, the WHO recommends adopting lower CER thresholds (€5–10/QALY) for AI tools targeting high-burden complications to balance accessibility and cost-effectiveness. Thailand’s National Health Security Office, for example, applied this threshold to reimburse the low-cost AI medication adherence app “DiaCare” (CER = €1.2/QALY) in 2023. By 2024, the app had been adopted by 800,000 T2DM patients, reducing medication non-adherence by 40% and saving €12 million in hospital admission costs (117), providing a context-adapted regulatory reference for resource-constrained regions.

#### 6.2.3 Infrastructure and workforce investment as policy prerequisites

The integration of AI and public health also requires a solid technical foundation and a high-quality talent team, with three key policy priorities: First, increase investment in digital infrastructure. Governments should allocate funds to build and upgrade public health digital infrastructure—including high-speed networks, cloud computing platforms, and secure data centers (162). For low-resource regions, priority should be given to resource input: advancing rural electrification, expanding access to low-cost internet (e.g., satellite internet in remote areas), and supporting offline syncing of AI tools to address connectivity gaps. A practical illustration of this priority is South Korea’s “Digital Health New Deal” (2022–2025), which invested $200 million in rural digital infrastructure and deployed 5G-enabled portable AI screening devices in 1,200 community health centers; these devices use edge AI to analyze finger-prick glucose data and retinal images, boosting the T2DM early detection rate in rural areas by 35% compared to 2021 (112).

Second, formulate professional talent training plans. Scholars such as Dimitra Panteli propose in their research to strengthen cooperation with universities and research institutions to cultivate interdisciplinary talents with knowledge of AI technology, data science, and public health. At the same time, through continuing education and training programs, improve the digital literacy and AI application capabilities of existing public health workers (11)—a model exemplified by Brazil’s Ministry of Health, which partnered with Microsoft in 2023 to provide free edge AI training to 3,000 primary care workers in the Amazon region; after 3 months of training, 92% of these workers could independently operate AI tools to optimize insulin dosages, reducing severe hypoglycemia cases by 28% in the target regions (29).

In addition, promote industry–academia–research cooperation. Policies should encourage collaboration between public health institutions, research institutes, and technology companies to jointly develop and deploy AI solutions tailored to public health needs (11) while providing policy incentives to support the innovative application of AI in the public health field.

#### 6.2.4 Ethical oversight, transparency requirements, and public participation mechanisms

**Figure 11:**
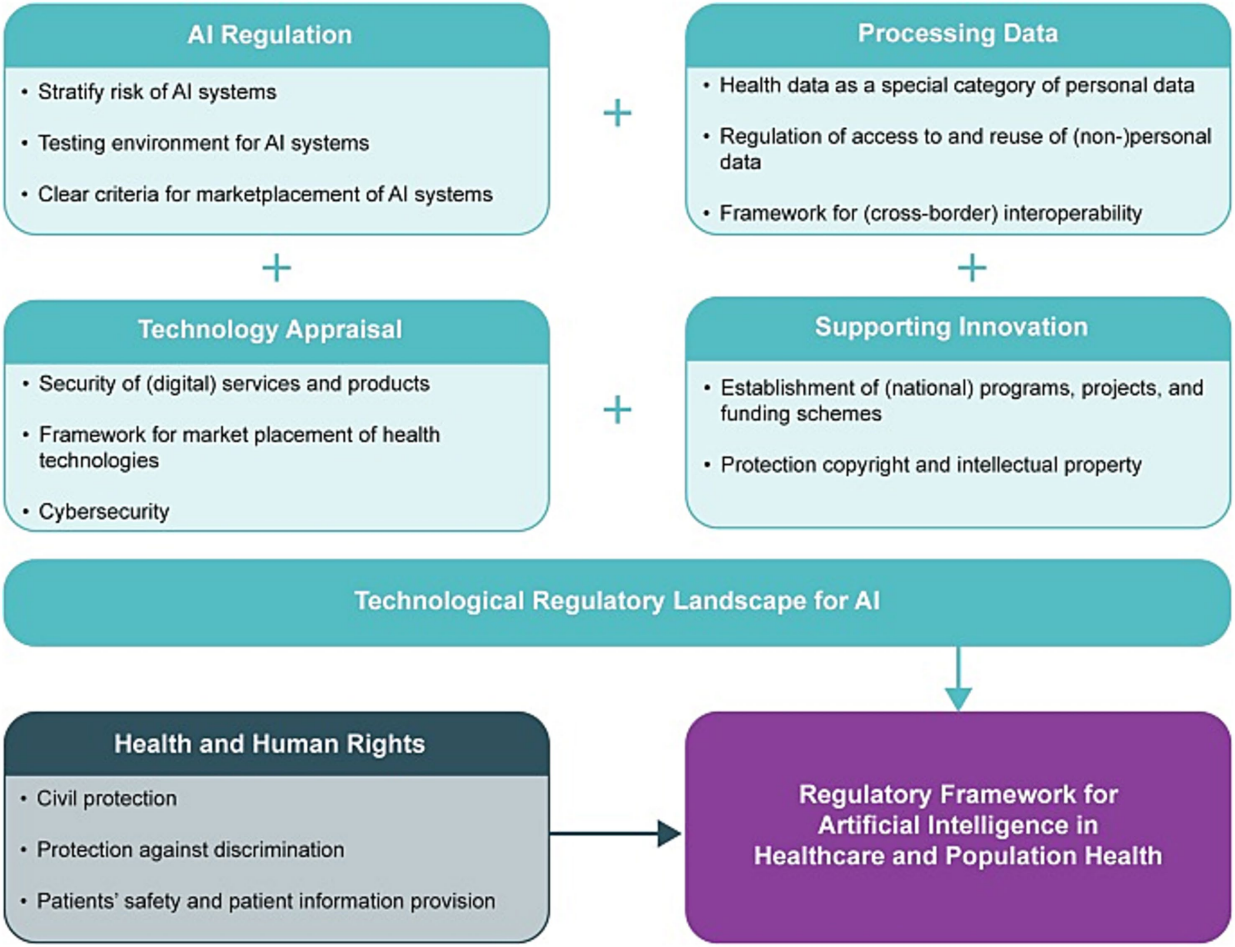
Regulatory framework for artificial intelligence in healthcare and population health. Figure adapted with permission from Schmidt et al. (159).

The application of AI in public health raises ethical issues such as algorithmic bias, fairness, and transparency. To address these issues and promote AI’s ethical, safe, and effective implementation in healthcare (while reducing risks), governments worldwide are establishing targeted regulatory frameworks—unlike broad digital health regulations, these frameworks are increasingly tailored to T2DM management needs, focusing on early screening, glucose control optimization, and complication prevention as core objectives (159). As shown in Figure 11, internationally, regulatory frameworks for AI in healthcare and population health are gradually taking shape: A prominent example is the EU’s “AI Act” (2024), which not only sets an overall regulatory framework for AI (including risk stratification, testing environments, and market access standards) but also explicitly classifies AI-driven diabetes management tools as “high-risk,” mandates ethnic group-specific bias audits, and is supplemented by legislation covering social, health, and human rights standards (159). A practical illustration of this Act’s impact is the German CGM analytics tool “GlucoAI”: Audits under the Act revealed it underestimated diabetic nephropathy risk in Turkish-German patients by 29% (due to unaccounted traditional dietary patterns like high legume intake), and retraining the algorithm to integrate ethnicity-specific dietary features improved its AUC in this subgroup from 0.67 to 0.81 (159).

To further strengthen the ethical governance of AI in public health, four key measures are critical: First, establish a multidisciplinary ethical review committee—comprising medical experts, ethicists, legal experts, social scientists, and AI specialists—to conduct comprehensive ethical reviews of AI applications, ensuring compliance with principles of fairness, non-discrimination, transparency, beneficence, non-maleficence, autonomy, and justice; these principles should guide AI designers and users throughout the system lifecycle (29, 106). Second, enhance algorithm transparency and interpretability, particularly for key decision scenarios (e.g., disease diagnosis and resource allocation), to enable users and regulators to understand AI decision bases and identify/correct biases; for algorithmic bias (rooted in skewed training data and harming marginalized groups like ethnic minorities, women, older adults, or low-socioeconomic populations), regulatory frameworks should mandate developers to mitigate bias via diverse data collection, fairness-aware algorithms, and continuous output monitoring (29, 106). Third, introduce public participation mechanisms (e.g., public consultations, hearings, and citizen committees) to solicit societal concerns and expectations regarding AI in public health, integrating public values into policy formulation to boost trust and social acceptance of policies (153). Finally, prioritize fairness for vulnerable groups (e.g., older adults, persons with disabilities, and low-income populations) in policymaking, ensuring AI does not exacerbate digital divides or health inequalities—for instance, incorporating multi-language support and auxiliary technologies when promoting digital health tools (162).

#### 6.2.5 Cross-sector + international cooperation for generalizable and fair evidence generation

T2DM is a global issue, and the development of AI and digital health technologies also has global characteristics. Currently, common policy silos and related challenges between different sectors seriously hinder the application of AI in the public health field and global governance and policies. Therefore, establishing effective cross-sectoral coordination mechanisms and encouraging international cooperation are urgent tasks.

First, establish an intersectoral policy coordination team composed of health, science and technology, legal, and ethical sectors, responsible for the formulation and evaluation of digital health and AI policies. Hold regular meetings to ensure policy coordination and consistency. Second, support large-scale research projects involving multiple countries, such as international multi-center clinical trials for T2DM, using AI to analyze data from different populations to improve the generalizability and fairness of AI models. At the same time, establish international digital health innovation centers to promote technology exchange and talent training, and actively participate in discussions on AI ethics and governance by international organizations such as the WHO and the United Nations Educational, Scientific and Cultural Organization (UNESCO), learning from international guidelines and best practices and integrating them into domestic policy formulation (29). Additionally, in south–south cooperation, Brazil has shared its low-cost AI T2DM management model (with a CER of €0.8 per QALY) with Peru. This model was integrated into Peru’s public health systems, delivering tangible economic and clinical value by saving approximately €300,000 annually for a cohort of 10,000 T2DM patients, with its successful scaling enabled by the sharing of key cost-effectiveness data (e.g., rates of complication reduction and long-term maintenance costs) between the two countries (29). In north–south partnerships, initiatives such as Microsoft’s AI for Health program exemplify effective technology transfer by providing free ML tools to LMICs, helping these regions bypass high upfront technology acquisition costs and accelerate the adoption of AI-driven T2DM management solutions (29).

In conclusion, promoting the integration of AI technology and public health policies requires a comprehensive regulatory framework that is multi-level, dynamically adjusted, and focuses on ethics and social fairness. It not only involves technical and legal aspects (including compliance with regulatory standards, data privacy protection, and accountability) but also relates to social trust and value orientation. By combining privacy-enhancing technologies (e.g., federated learning, homomorphic encryption, and blockchain) with compliant frameworks, healthcare systems can foster an environment that encourages innovation while safeguarding patient trust (159). Through the implementation of the above policy recommendations, it is expected to ensure that AI plays a transformative role safely in the public health field.

## 7 Conclusion

### 7.1 Prospects of AI and digital health

#### 7.1.1 Application prospects of AI and digital health tools in T2DM management

(1) Mechanism prediction and early intervention in basic research: AI can identify complex patterns from large-scale biomedical data, enabling deeper insights into T2DM mechanisms. By integrating multi-omics data (e.g., genomics, proteomics, metabolomics, and microbiomics), AI supports system-level modeling of T2DM pathogenesis and facilitates the discovery of molecular pathways and candidate biomarkers (11). In parallel, AI can leverage EHRs, wearable data, and lifestyle information to develop individualized risk prediction models that identify high-risk individuals before clinical onset, thus enabling earlier intervention and prevention strategies (163). Given that T2DM is frequently accompanied by complications, predictive models can also support anticipatory risk stratification and preventive clinical actions, reducing long-term patient burden.

(2) Personalized treatment and precise management in clinical applications: As shown in Table 1, AI approaches differ in clinical benefits and scalability—some methods (e.g., XGBoost and edge AI) demonstrate practical value for deployment, whereas others (e.g., digital twins) remain largely confined to academic contexts. For personalized drug selection and dose adjustment, AI can integrate genetic background, clinical characteristics, lifestyle factors, and medication response patterns to recommend more suitable hypoglycemic regimens, potentially improving efficacy and reducing adverse events. For intelligent glucose management, AI-enabled digital health tools (e.g., smart insulin pumps and CGM systems) can support real-time monitoring, prediction of hypo/hyperglycemic events, and data-driven insulin titration to improve glucose stability and TIR (163). In addition, remote monitoring and digital follow-up can enhance care accessibility and continuity—particularly for patients in remote areas or those with mobility impairments—by enabling timely treatment adjustments and more efficient service delivery (11).

(3) Fairness and accessibility in public health: By analyzing multi-source population data and identifying high-risk groups, AI can support earlier public health interventions and more efficient resource allocation, which is particularly valuable in resource-constrained settings. However, equitable benefits require systematic attention to algorithmic bias and model generalizability across racial, ethnic, and socioeconomic groups. Digital health tools (e.g., mHealth apps and wearable devices) can extend real-time monitoring and personalized feedback beyond traditional clinic settings, strengthening self-management and improving access for underserved populations, including those in remote regions or with limited mobility.

#### 7.1.2 Importance of interdisciplinary cooperation

To maximize the impact of AI and digital health tools in T2DM management—and to ensure their responsible integration into public health systems—interdisciplinary cooperation is essential. Policymakers and technical experts should jointly align innovation with regulatory compliance, data security, and ethical governance (161), while ensuring solutions are implementable and interoperable in real-world systems (11). Clinicians and medical researchers can define clinically meaningful endpoints and guide validation, whereas data scientists translate these needs into robust prediction and decision-support models through ML and large-scale data processing (164). Ethicists and social scientists are critical for addressing fairness, privacy, and societal implications to avoid reinforcing health inequities. Public health institutions and industry partners can accelerate translation through PPPs, supporting evaluation, scale-up, and cost-effectiveness in large-scale deployment (11). Finally, patient and public participation via co-creation/co-design should be embedded early to improve usability, acceptability, and adherence, and to strengthen the fairness and practicality of clinical evaluation.

In conclusion, AI and digital health tools can reshape T2DM management across the continuum from mechanism discovery and early risk detection to personalized therapy and scalable remote care. Yet real-world impact depends not only on algorithmic performance but also on governance, equity, and implementation capacity. Therefore, translating this potential into population-level benefit requires coordinated action among policymakers, technologists, clinicians, data scientists, ethicists, social scientists, industry partners, and patients to build an ecosystem that is effective, safe, and equitable.

### 7.2 Future research directions and policy recommendations

To ensure that AI technology can benefit T2DM patients worldwide, especially those in LMICs, future research directions must prioritize low-resource adaptation and focus on three core areas: data privacy/security, algorithmic fairness/bias mitigation, and policy formulation for equitable digital health.

#### 7.2.1 Data privacy and security: protecting sensitive health data in AI-driven T2DM management

As AI adoption deepens, large volumes of sensitive health data are collected and processed, making privacy and security foundational for sustainable deployment. Future research should advance privacy-preserving learning and analytics that reduce reliance on centralized raw data sharing. Key directions include: (1) FL to support decentralized training and reduce data leakage risks; and (2) Differential Privacy to protect individual identities via controlled noise injection while preserving utility for AI modeling.

From a policy perspective, countries should strengthen data governance frameworks by clarifying legal and ethical requirements for data collection, use, storage, sharing, and destruction, with special attention to feasibility in low-resource settings. In addition, investments in cybersecurity infrastructure and operational safeguards are needed to reduce cyberattack vulnerability and unauthorized access. Finally, improving data transparency—so patients understand how data are used—and ensuring rights to access, correction, and deletion can enhance trust and long-term adoption of digital health tools.

#### 7.2.2 Algorithmic fairness and bias mitigation: ensuring equitable AI for diverse T2DM populations

T2DM populations vary across genetics, lifestyle, and socioeconomic contexts; models trained on non-representative data may produce biased predictions and recommendations, worsening health inequities. Future research should (1) develop debiasing methods that detect and mitigate systematic disparities in model performance; (2) build more diverse, multimodal datasets representing heterogeneous global populations to reduce subgroup-specific failure; and (3) establish algorithm audit mechanisms and independent evaluation toolkits to support ongoing monitoring of fairness, transparency, and interpretability (29).

Policy efforts should require transparency around model design principles, training data sources, intended use populations, and potential biases—particularly for tools informing high-stakes medical decisions (29). In parallel, interdisciplinary ethics review structures should evaluate potential impacts across patient subgroups before large-scale rollout and provide actionable recommendations for risk mitigation (29).

#### 7.2.3 Policy transformation for global AI-enabled T2DM management

To unlock AI’s full potential, public health policies must adapt to digital disruption through targeted and inclusive measures: (1) investing in inclusive digital infrastructure in resource-poor and rural areas to enable reliable Internet access and basic digital health service capacity; (2) strengthening digital health education and workforce training, including patient-facing tool literacy and clinician training on AI-supported workflows, while ensuring multi-language and accessibility functions to address diverse needs; (3) establishing PPPs to coordinate governments, public health agencies, technology companies, and academia for the development, testing, and scaling of AI-driven solutions; (4) integrating AI and digital health tools into national/regional strategies with clear roadmaps, governance structures, and resource allocation plans spanning prevention, diagnosis, treatment, and long-term management.

#### 7.2.4 Critical unresolved bottlenecks: beyond “more data, better models”

Beyond technical optimization, future research must adopt a context-aware lens to address persistent implementation gaps:

(1) Frugal AI for LMICs: shift from replicating HIC technologies to co-designing low-resource-adapted solutions. Prioritize clinical utility under constrained and incomplete data conditions (e.g., sparse EHRs and limited wearable coverage) rather than maximal model complexity.
(2) Behavioral science integration: evaluate how cultural norms and socioeconomic constraints (e.g., family-centered care patterns and work conditions limiting CGM adherence) shape real-world effectiveness, treating these factors as core determinants rather than externalities.
(3) Long-term cohort and pragmatic evaluations: conduct 5–10 year follow-up studies to quantify impacts on hard outcomes beyond HbA1c, including disability-free survival and health-related quality of life—particularly relevant where indirect costs such as productivity loss account for substantial disease burden (8).
(4) Context-specific regulatory pathways: adopt risk-based evaluation models—low-risk tools (e.g., medication reminders) may undergo abbreviated review, whereas high-risk tools (e.g., insulin dose optimizers) should require multi-center validation across diverse populations.
(5) Structural inequality safeguards: audit whether AI-driven resource allocation systematically disadvantages underserved groups (e.g., urban–rural gaps due to data availability) and develop countermeasures (e.g., weighting strategies that prioritize underserved regions).

#### 7.2.5 Evidence-based scaling

Scaling should prioritize AI tools with demonstrated clinical benefits (e.g., HbA1c reduction and TIR improvement) and avoid overinvestment in approaches that repeatedly fail to generalize across populations (e.g., persistently unadopted models despite high internal accuracy). Prospective validation should mandate reporting of clinically meaningful endpoints (HbA1c, TIR, hypoglycemia) rather than prediction metrics alone, alongside systematic documentation of failures to inform iterative improvement and prevent repeated implementation pitfalls.

Collectively, these research priorities and policy actions provide a pragmatic pathway for translating AI-enabled digital health into more precise, personalized, accessible, and equitable T2DM management worldwide—while safeguarding privacy and fairness and strengthening global public health outcomes.
